# Traditional Chinese Nootropic Medicine *Radix Polygalae* and Its Active Constituent Onjisaponin B Reduce β-Amyloid Production and Improve Cognitive Impairments

**DOI:** 10.1371/journal.pone.0151147

**Published:** 2016-03-08

**Authors:** Xiaohang Li, Jin Cui, Yang Yu, Wei Li, Yujun Hou, Xin Wang, Dapeng Qin, Cun Zhao, Xinsheng Yao, Jian Zhao, Gang Pei

**Affiliations:** 1 State Key Laboratory of Cell Biology, Institute of Biochemistry and Cell Biology, Shanghai Institutes for Biological Sciences, Chinese Academy of Sciences, Shanghai, China; 2 Graduate School, University of Chinese Academy of Sciences, Chinese Academy of Sciences, Shanghai, China; 3 Institute of Traditional Chinese Medicine and Natural Products, College of Pharmacy, Jinan University, Guangzhou, China; 4 Translational Medical Center for Stem Cell Therapy, Shanghai East Hospital, School of Medicine, Tongji University, Shanghai, China; 5 School of Life Science and Technology, Collaborative Innovation Center for Brain Science, Tongji University, Shanghai, China; Torrey Pines Institute for Molecular Studies, UNITED STATES

## Abstract

Decline of cognitive function is the hallmark of Alzheimer’s disease (AD), regardless of the pathological mechanism. Traditional Chinese medicine has been used to combat cognitive impairments and has been shown to improve learning and memory. *Radix Polygalae* (RAPO) is a typical and widely used herbal medicine. In this study, we aimed to follow the β-amyloid (Aβ) reduction activity to identify active constituent(s) of RAPO. We found that Onjisaponin B of RAPO functioned as RAPO to suppress Aβ production without direct inhibition of β-site amyloid precursor protein cleaving enzyme 1 (BACE1) and γ-secretase activities. Our mechanistic study showed that Onjisaponin B promoted the degradation of amyloid precursor protein (APP). Further, oral administration of Onjisaponin B ameliorated Aβ pathology and behavioral defects in APP/PS1 mice. Taken together, our results indicate that Onjisaponin B is effective against AD, providing a new therapeutic agent for further drug discovery.

## Introduction

Alzheimer’s disease is a complex and currently incurable age-related neurodegenerative disease and is highly prevalent in aged cohorts worldwide [1]. It is the most common late-age mental failure in humans and currently exerts great economic and political pressure on modern society. Aβ deposition, tau tangles and cognitive degeneration are the hallmarks of the disease [2], therefore reducing Aβ production and improving cognitive function has be considered as an effective disease- and symptom-modifying therapeutic strategy.

The amyloid cascade hypothesis is supported by accumulating studies based on cell culture and animal experiments [3]. In amyloid hypothesis, the maturation, processing and degradation of APP and the consequential production and clearance of Aβ initiate AD pathogenesis [4]. APP can be sequentially cleaved by BACE1 and γ-secretase [5, 6] and finally yield Aβ species. This makes BACE1 and γ-secretase key-players in AD pathogenesis. In brains of Alzheimer’s disease patients, large amounts of Aβ are produced and aggregated, mainly in the hippocampus and prefrontal cortex, causing neuronal death and impairment of cognitive function [7, 8]. Thus, for the past two decades, to identify a solution for this devastating disease, researchers have focused on modulating BACE1 or γ-secretase activities. The cellular Aβ level can be reduced, and cognitive function impairments have been shown to be ameliorated in transgenic AD model mice treated with secretase inhibitors or modulators [9]. However, treatments with these compounds have been discontinued because of severe adverse effects in recent clinical trials [10, 11]. Aside from functions in Aβ generation, BACE1 and γ-secretase are also involved in multiple physiological processes including cell adhesion and Notch signaling. These accumulating evidences suggest that the strategy of directly inhibiting the enzymatic activity of BACE1 and γ-secretase may need to be optimized. APP and its proteolytic products undergo degradation via protein degradation pathways [12–14]. The proteasome is the major organelle for protein degradation in cells [15], and cleavage through this pathway reduces Aβ production [16–18]. Furthermore, our previous work has shown that interfering with the interaction between BACE1 and γ-secretase, thereby blocking the sequential process and reducing Aβ production, may have some advantages in modifying the disease [19].

Traditional Chinese medicine has long been used to treat dementia [20–23]. Among those historically used herbal drugs, *Radix Polygalae* (RAPO) has been demonstrated to exhibit nootropic activity [20, 24, 25]. Moreover, our previous data regarding the “Smart soup” containing *Rhizoma Acori Tatarinowii*, *Poria cum Radix Pini* and *Radix Polygalae* showed systematic beneficial effects against AD and RAPO function to decrease Aβ production [24, 26]. Herein, we explored the major component(s) of RAPO and the related underlying mechanism.

## Materials and Methods

### Ethics Statement

All animal experiments were performed according to the National Institutes of Health Guide for the Care and Use of Laboratory Animals. The animal protocols were approved by the Biological Research Ethics Committee, Shanghai Institutes for Biological Sciences, Chinese Academy of Sciences. Effort was made to minimize animal pain and discomfort. The IACUC approved this research under the approval number SIBCB-NAF-14-002-S309-015.

### Animal

The APPswe/PS1ΔE9 (APP/PS1) double-transgenic mice (JAX Stock No. 004462) brought from Jackson Laboratory express a chimeric mouse/human amyloid precursor protein (Mo/HuAPP695swe) and a mutant human Presenilin 1 (PS1ΔE9). The mice were maintained and genotyped according to the Jackson Laboratory guidelines.

### Drug administration

The transgene-negative wild-type (WT) littermates were used as gender- and age-matched controls. RAPO-1-3 or Onjisaponin B was dissolved in vehicle (50% PEG400 in H_2_O). APP/PS1 and WT mice were chronically administered 200 μl of Onjisaponin B (1 mg/ml), RAPO-1-3 (15 mg/ml) or vehicle per 20 g body weight by oral gavage once per day from 4 to 7 months of age (n = 12 to 16 mice per group).

### Morris water maze

The Morris water maze analysis was performed as previously reported [27], and the animals were randomly numbered among genotypes and grouped for the test. The apparatus was a 120-cm-diameter circular pool filled with water containing small white plastic particles, with cues of four different shapes posted on four directions of the inner pool wall. The water temperature was maintained at 23.0 ± 0.5°C and the room temperature at 25.0 ± 0.5°C during the whole procedure. A transparent platform 11 cm in diameter was placed 1 cm below the water surface at a fixed position in the target quadrant. The training consisted of 4 trials per day for 7 consecutive days. On day 4, probe trials were conducted after the fourth training trial. On day 8, a single round of probe trial was performed. An automated tracking system (Ethovision XT software) was used to monitor the mouse swimming paths and other parameters.

### Immunohistochemistry and image analysis

The mice were anesthetized and transcardially perfused with phosphate-buffered saline (PBS) buffer and then with 4% paraformaldehyde (PFA). Brain cryo-sections (30 mm thick) were prepared and immunostained using 6E10 for amyloid plaques and GFAP for astrocytes. Images were captured using a Carl Zeiss Z1 microscope (Zeiss). Quantification was performed using Image-Pro Plus 5.1 software (Media Cybernetics). Ten to fifteen coronal sections were analyzed per mouse.

### Compounds, reagents and antibodies

Onjisaponin B (purity > 98%) was purchased from Biopurity, and RP granules were purchased from Jiangyin Tianjiang Pharmaceutical Co., Ltd. L-685,458 (purity > 96%) and (S)-(+)-ibuprofen (purity > 99%) were purchased from Sigma, DAPT (purity > 99%) was purchased from Selleck and BACE1 inhibitor IV (purity > 98%) was purchased from Calbiochem. E2012 was synthesized by Ginkgo Pharma. MG132 (purity > 97%) was purchased from Selleck and lactacystin (purity > 98%) from Santa Cruz. CellTiter-Glo was purchased from Promega. Fugene HD and Effectene Transfection Reagent were purchased from Roche and QIAGEN, respectively. Immunoblotting was performed with the following antibodies: anti-ADAM10 (a Disintegrin and metalloproteinase domain-containing protein 10) C-term (Sigma); anti-PS1 N (1–65) (EMD); anti-BACE1 N-term (Abgent); anti-APP-CTF (Sigma); anti-Flag (Sigma); anti-sAPPα (secreted Amyloid Precursor Protein-α) (IBL); anti-sAPPβ (secreted Amyloid Precursor Protein-β) (IBL); anti-HA (Sigma); anti-c-Myc (Santa Cruz); anti-NICD (Notch intracellular domain) (Cell Signaling); anti-E-Cadherin-CTF (BD Transduction Laboratories); anti-APLP1 (APP-like protein 1) C-Terminal (643–653) (Calbiochem). Secretase activity assays were performed with anti-Aβ40 (EMD Millipore) and anti-Aβ (82E1, IBL). Immunohistochemistry was performed with anti-Aβ, 1–16, 6E10 (Covance) and anti-GFAP (Glial fibrillary acidic protein) (Dako).

### Cell culture and plasmids

HEK293T, HEK293 and A431 cells were previously purchased from ATCC, and HEK293MSR cells were a kind gift from Sanofi-Aventis Research and Development. All cell lines were maintained under the same condition as described previously [19]. In detail, HEK293T, HEK293MSR, HEK293/APPswe and A431 were cultured in Dulbecco’s modified Eagle’s medium (DMEM) with 10% (w/v) heat-inactivated fetal bovine serum in a humidified incubator with 5% CO2/95% air (v/v) at 37°C. HEK293 cells were cultured in MEM under the same condition. The Swedish mutant form of APP was transfected into HEK293 using Fugene HD (Roche) following the manufacturer’s instructions. A cell line stably expressing Swedish mutant APP was established in the presence of 1 mg/ml G418. Penicillin-Streptomycin solution (Life Technologies) was added according to the manual. All constructs were the same as reported previously and were verified by sequencing [19, 28].

### ELISA for Aβ

HEK293/APPswe cells were treated with chemicals at the indicated concentrations and durations. The conditioned medium was then collected and subjected to a sandwich ELISA for the total Aβ level. Human Aβ40 and Aβ42 in APP/PS1 mouse brains were extracted as previously reported [29] and measured with human Aβ ELISA kits according to the manufacturer’s guidelines. ELISA kits for total human Aβ, human Aβ40 and human Aβ42 were obtained from ExCell Bio.

### *In vitro* BACE1 and γ-secretase assays

Total membrane fractions were extracted from 293T cells or APP/PS1 mouse brain and used in ELISA-based secretase assays or fluorogenic substrate assays to measure BACE1 or γ-secretase activity. Fluorogenic substrate assays and the ELISA-based γ-secretase assay were carried out as previously reported [30, 31]. For the ELISA-based BACE1 assay, membrane fractions of HEK293T cells were collected after lysis in buffer A. Supernatants containing 20 mg protein were centrifuged at 25000 *g* for 1 hour. The resulting membrane pellets were then resuspended in BACE1-assay buffer (50 mM sodium acetate, pH 4.5 and 0.5 mM biotinylated APP-TM peptide). After being incubated at 37°C for 30 minutes, the reaction mixtures or biotinylated standard peptides DK-16 were neutralized by Tris-Na_2_HPO_4_ buffer and added into streptavidin-coated 96-well plates (Pierce) and incubated at room temperature for one hour. Anti-Aβ 82E1 antibodies (IBL) were added to the plate and then incubated for another hour. After three washes, horseradish peroxidase-labeled anti-mouse antibodies were added, and the plates were incubated for one hour. Ultra-TMB (Pierce) was used as the substrate for horseradish peroxidase and the absorbance at 450 nm was recorded. Concentrations of samples were calculated according to standard curves. APP-TM peptide (amino acid sequence: SGLTNIKTEEISEVNLDAEFRHDSGYEVHHQK-biotin) and DK-16 (amino acid sequence: DAEFRHDSGYEVHHQK-biotin) were synthesized by GL Biochem.

### Secretase substrate processing assays

HEK293/APPswe cells or HEK293T cells transiently transfected with myc-NotchΔE or PSGL1-HA were treated with chemicals for 4 hours, and membrane fractions or total lysates were analyzed for APP-CTF, NICD and PSGL1-CTF levels. Culture medium from HEK293/APPswe cells was subjected to Western blotting analysis for sAPPα and sAPPβ and to ELISA analysis for total Aβ. The *in vitro* C99 assay was carried out as described previously [32]. The *in vitro* APP processing assay was performed using the membrane fraction of HEK293/APPswe cells incubated with the indicated chemicals *in vitro* at 37°C for 2 hours and then subjected to Western blot for APP-CTF analysis. Membrane fractions or total lysates of mouse brain were extracted and subjected to Western blotting analysis for ADAM10, BACE1, PS1, APLP1 and its CTF, and E-Cadherin CTFs. Flotillin or Actin was blotted as a loading control.

### Statistical analysis

All experiments were repeated at least three times. All data are presented as the mean ± s.e.m. and analyzed using GraphPad Prism 6.01 (San Diego, CA, USA). The unpaired Student’s *t*-test was used for comparisons of two groups. Group differences were analyzed with one-way analysis of variance (ANOVA). The results of Morris water maze hidden platform training were analyzed using two-way ANOVA. Differences were considered significant when *p* < 0.05.

## Results

### Systematic fractionation identifies RAPO-1-3 as the active fraction of RAPO that reduces Aβ production

**Fig 1:**
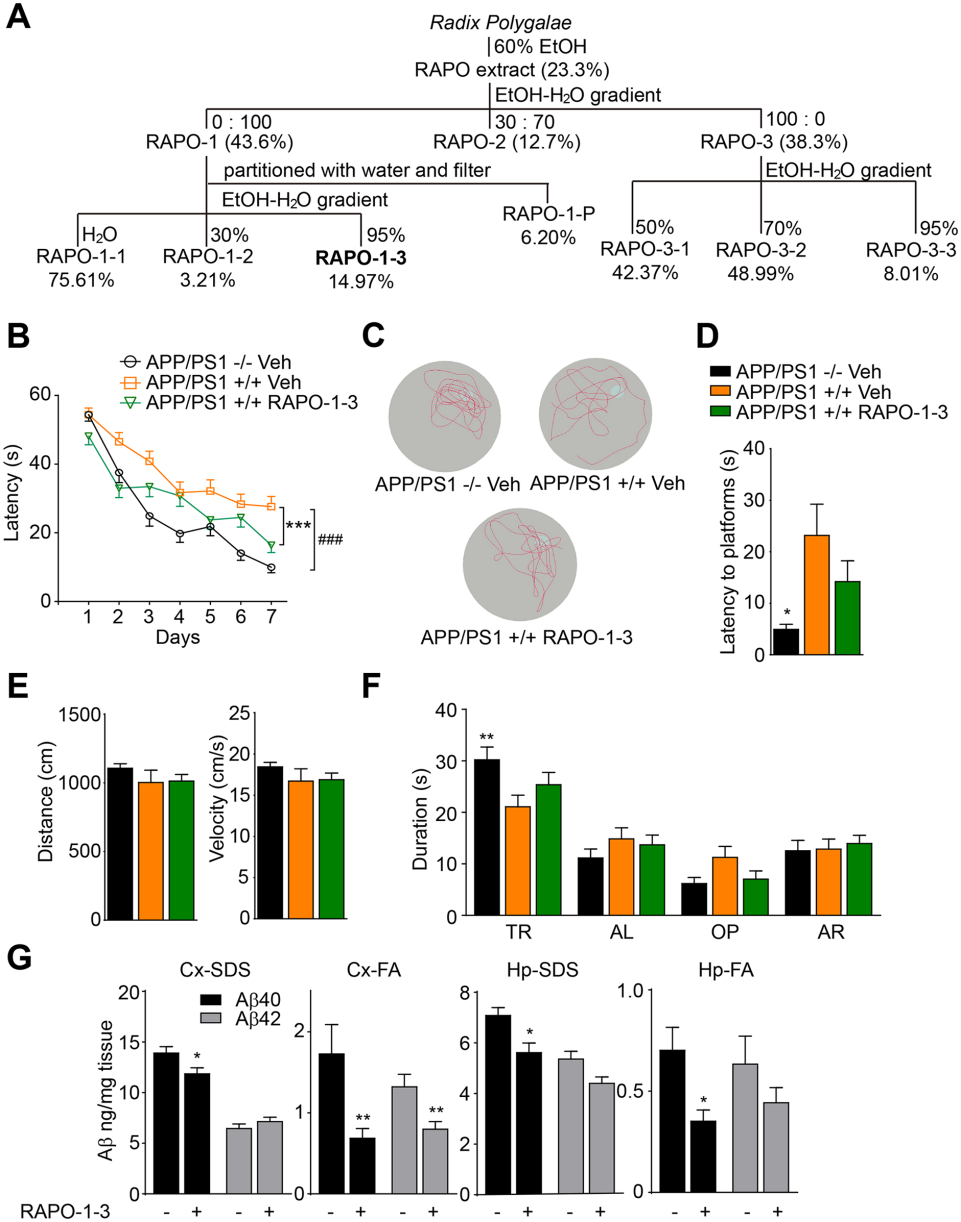
Systemic fractionation identifies RAPO-1-3 as the active fraction of RAPO that reduces Aβ production. (A) The extraction and fractionation scheme of RAPO. (B) RAPO-1-3 significantly reverses the spatial memory deficit of AD mice. (C) Representative tracks of each group of mice in probe trial test at day 8. (D) The latency to platform in probe trial for each group of mice at day 8. (E) No differences in the swimming distance and velocity among the groups. (F) The time spent by mice in the target quadrant. (G) SDS-soluble and FA-soluble Aβ40 and Aβ42 levels in the mouse hippocampi and cortices were measured by sandwich ELISA and normalized to control. Data are presented as the mean ± s.e.m. * *p* < 0.05, ** *p* < 0.01 and *** *p* < 0.001. Two-way ANOVA with Bonferroni's multiple comparison test (B, F), one-way ANOVA with Bonferroni's multiple comparison test (D, E) and two-tailed *t*-test (G).

In our previous study, we showed that *Radix Polygalae* reduced Aβ generation *in vivo* and *in vitro* [26]. To identify the active components, we first separated RAPO into 3 fractions using an ethanol-water gradient (Fig 1A). The concentrations of the fractions were calculated according to the yield and are presented as weight/volume (w/v) of original raw material. The concentrations of the different RAPO fractions applied to HEK293/APPswe cells are 0.1, 0.3 and 1 mg/ml (relative RAPO concentration). This relative concentration is converted based on the yield of each fractionation step. For example, according to the fractionation scheme, RAPO-1 is composed of 10.1588% dry weight of RAPO (43.6% × 23.3% = 10.1588%), and 1 mg/ml (relative to RAPO) of RAPO-1 is equivalent to 0.1 mg/ml (actual concentration). We then treated HEK293/APPswe cells with various concentrations of each fraction. HEK293/APPswe cells stably overexpress human APP protein carrying a Swedish mutant (K595N/M596L) and show elevated Aβ secretion [33]. Medium from HEK293/APPswe cells treated with RAPO fractions was collected to detect the total Aβ level using a sandwich ELISA. Meanwhile, cells were lysed and subjected to the CellTiter-Glo assay to evaluate cell viability. The 8-hour treatment with RAPO-1 or RAPO-2 did not affect cell viability, whereas RAPO-3 reduced cell viability to 48% (S1A Fig). Compared to vehicle treatment, treatment with either RAPO-1 or RAPO-3 markedly reduced the Aβ level in the conditioned medium, by 48% and 90%, respectively (S1A Fig). To test whether the Aβ-reducing activity and cytotoxicity might be separated, we further fractioned RAPO-1 and RAPO-3 into three sub-fractions (Fig 1A). The cell viability was severely compromised upon RAPO-3-2 treatment, decreasing by 68% (S1B Fig), whereas the fraction RAPO-1-3 reduced Aβ generation without obvious cytotoxicity (S1B Fig). These results indicate that RAPO-1-3 is the representative fraction of RAPO that reduces Aβ production without affecting cell viability. Therefore, we focused on RAPO-1-3 in our further studies.

We then tested the *in vivo* efficacy of RAPO-1-3. It has been reported that APP/PS1 double transgenic mice begin to display accumulated Aβ plaque deposition in the hippocampus and cortex, as well as age-dependent deficits in cognitive function at 6 months of age [34–36]. We chronically administered RAPO-1-3 to APP/PS1 mice. Gender- and age-matched APP/PS1 transgenic mice and their transgene-negative littermates were grouped and treated with RAPO-1-3 (0.15 g/kg/day) or Vehicle (50% PEG400 in distilled water) by oral gavage. During the drug administration, animals’ body weights were recorded. The body weights in the RAPO-1-3 group showed no significant change compared to that of the vehicle group (data not shown), suggesting that there was no obvious toxic effect of the treatment. Three months later, the Morris Water Maze analysis was performed to evaluate the spatial learning and reference memory of the animals [27, 37, 38]. There was no obvious difference among animals in swimming distance or velocity (Fig 1E), implying that RAPO-1-3 treatment did not change mouse locomotor activity. As shown in Fig 1B, consistent with previous reports, APP/PS1 mice exhibited significantly impaired learning and memory ability compared to their wild-type littermates in the hidden platform phase (Veh APP/PS1 -/- vs. Veh APP/PS1 +/+, *p* < 0.0001) [36]. Interestingly, these spatial learning and memory deficits of APP/PS1 mice were ameliorated by chronic administration of RAPO-1-3 (RAPO-1-3 APP/PS1 +/+ vs. Veh APP/PS1 +/+, *p* < 0.0001). During the probe trial at day 8, the mice treated with RAPO-1-3 took less time to reach the position of the platform (RAPO-1-3 APP/PS1 +/+ vs. Veh APP/PS1 +/+, *p* = 0.2479) (Fig 1D), spent more time in the target quadrant (RAPO-1-3 APP/PS1 +/+ vs. Veh APP/PS1 +/+, *p* = 0.2116) (Fig 1F), and crossed more frequently within the platform area (Fig 1C). These data indicate that RAPO-1-3 effectively ameliorates the spatial learning and reference memory deficiency of APP/PS1 transgenic mice. Soluble Aβ oligomers are deleterious and correlate to cognitive deficits in Alzheimer’s disease [39, 40]. Hence, SDS-soluble and FA-soluble Aβ40 and Aβ42 in mouse cortices and hippocampi were quantified by sandwich ELISA. SDS-soluble Aβ40 and Aβ42 were moderately reduced in the cortex and hippocampus, while FA-soluble Aβ40 and Aβ42 were significantly reduced (Fig 1G). These data indicate that RAPO-1-3 functions as the active fraction of RAPO that reduces Aβ production *in vivo* and *in vitro*, as well as attenuating the learning and memory deficits in APP/PS1 mice.

### RAPO-1-3 and Onjisaponin B reduce Aβ production without affecting BACE1 or γ-secretase activity

**Fig 2:**
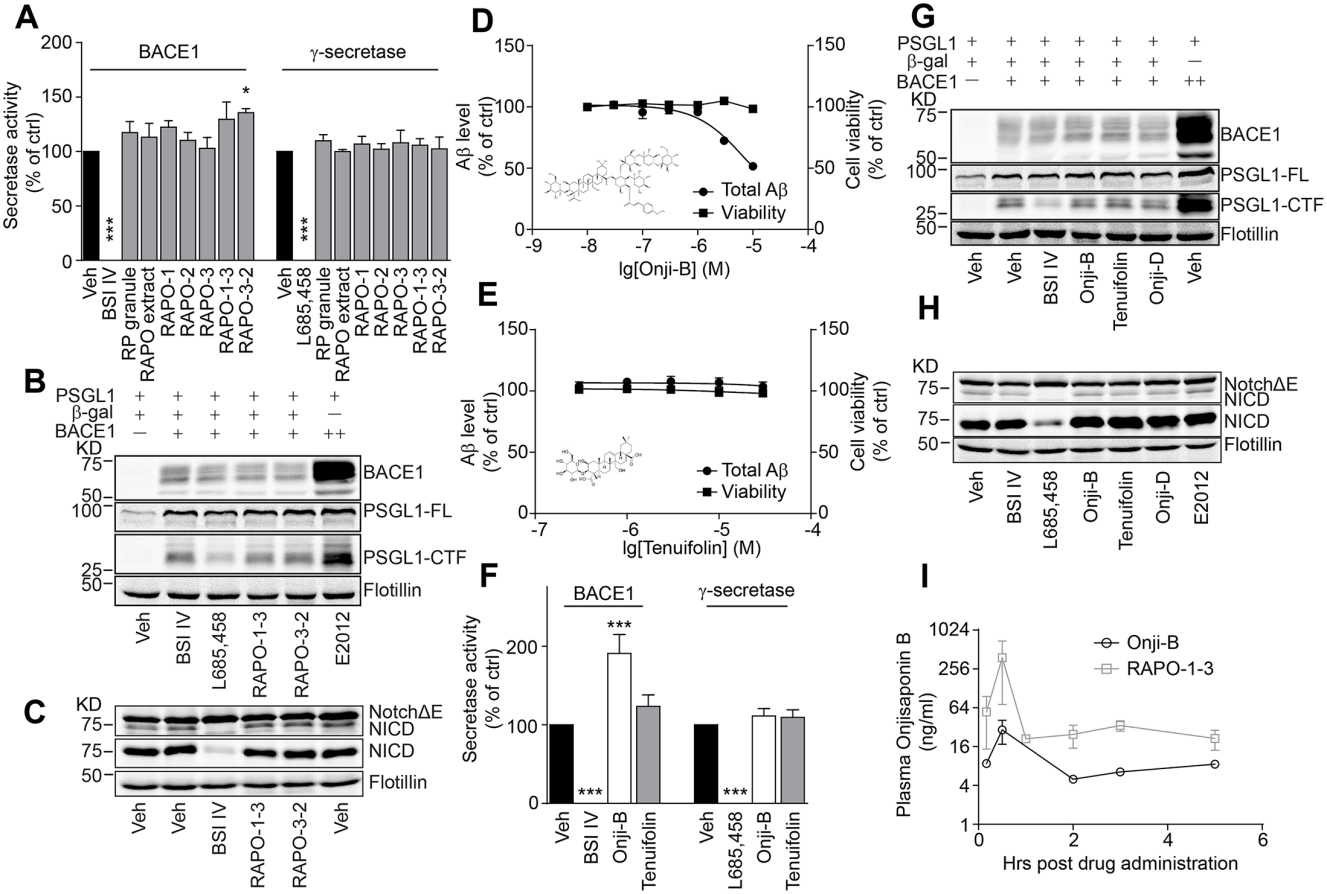
RAPO-1-3 and Onjisaponin B reduce Aβ generation without affecting the enzymatic activity of BACE1 or γ-secretase. (A) RAPO fractions (1 mg/ml) do not alter the activity of BACE1 or γ-secretase. (B-C) Neither RAPO-1-3 nor RAPO-3-2 (1 mg/ml) changes the processing of PSGL1 by BACE1 (B) or of Notch by γ-secretase (C). Onjisaponin B (D) but not Tenuifolin (E) reduces Aβ production without inhibiting BACE1 or γ-secretase (F) activity. (G-H) Neither Onjisaponin B nor Tenuifolin (10 μM) alters the processing of PSGL1 by BACE1 (G) or of Notch by γ-secretase (H). (I) Onjisaponin B concentration within mouse plasma after the acute administration of RAPO-1-3 or Onjisaponin B. In the figures, Onji-B stands for Onjisaponin B. Data are presented as the mean ± s.e.m. * *p* < 0.05, ** *p* < 0.01 and *** *p* < 0.001. One-way ANOVA with Bonferroni's multiple comparison test (A, F).

Aβ is produced by sequential processing of APP by BACE1 and γ-secretase [5, 41–44]. We next explored the activity of these two secretases. Using an ELISA-based secretase activity assay [45], we tested whether these active fractions directly inhibited secretase activity. As shown in Fig 2A, while BACE1 inhibitor IV (BSI IV) significantly inhibited BACE1 activity, and γ-secretase inhibitor (GSI) L685458 inhibited γ-secretase activity, RAPO fractions showed no significant effect on either secretase (Fig 2A). We further monitored the processing of other BACE1 and γ-secretase substrates in the presence of RAPO-1-3. P-selectin glycoprotein ligand-1 (PSGL1), a high-affinity counter-receptor for P-selectin that is responsible for tethering myeloid cells and stimulating T lymphocytes to activate platelets or endothelia, is another substrate of BACE1 [46, 47]. We investigated whether PSGL1 cleavage is affected by RAPO-1-3 using western blotting. The abundance of the C-terminal fragment of PSGL1 was significantly decreased in the presence of BSI IV and increased with transient overexpression of BACE1 as reported previously [47]. However, neither RAPO-1-3 nor RAPO-3-2 treatment altered the PSGL1 processing pattern (Fig 2B). Moreover, NICD level was markedly reduced by L685458, while the treatment with BSI IV or RAPO fractions did not interfere with the NotchΔE cleavage pattern (Fig 2C). These data suggest that RAPO-1-3 harbors Aβ-reducing activity and shows no direct inhibitory effects on the activities of BACE1 or γ-secretase.

**Fig 3:**
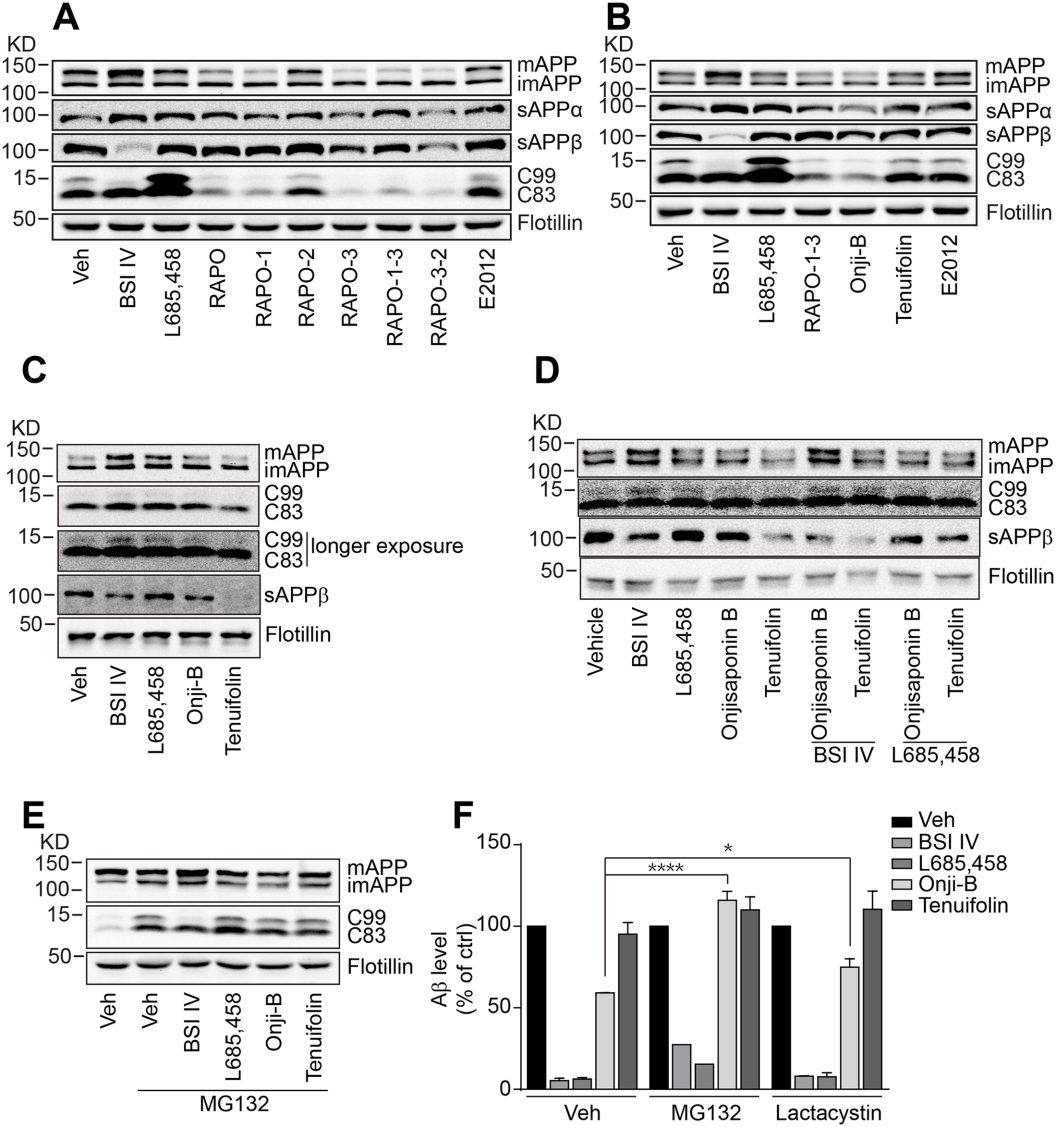
Onjisaponin B promotes APP degradation. (A-B) RAPO, RAPO fractions (1 mg/ml) (A) and Onjisaponin B (10 μM) (B) reduce mature APP levels in HEK293-APPswe cells. (C) Generation of sAPPβ in the presence of 10 μM Onjisaponin B or Tenuifolin in BACE1-assay buffer. (D) Mature APP was accumulated in detergent-soluble membrane fractions treated with 10 μM BSI IV together with Onjisaponin B. (E-F) The proteasome inhibitor MG132 (10 μM) prevents the reduction of mature APP (E) and Aβ generation (F) by Onjisaponin B. (F) The proteasome inhibitor lactacystin (20 μM) partially blocks the Aβ reduction by Onjisaponin B. Data are presented as the mean ± s.e.m. * *p* < 0.05, ** *p* < 0.01 and *** *p* < 0.001. Two-way ANOVA with Bonferroni's multiple comparison test (F).

To further identify the active constituents in RAPO-1-3, HPLC analysis was performed (data not shown). Several major chemical constituents were enriched in the RAPO-1-3 fraction. For detailed inspection, we performed UPLC-ESI-MS. Acyl saponins that possess the same saponin aglycone, similar glycosyl, and substituted groups account for approximately 12.8% of RAPO-1-3 (S2A–S2C Fig). Among them, Onjisaponin B was the relative abundant and characteristic one. The crude RAPO-1-3 extracts contained ~2.4% (w/w) Onjisaponin B according to the chromatogram of RAPO-1-3 and UPLC-Q/TOF-MS chromatogram of the pure standard compound (S2D–S2F Fig and S2 Table). In fractions adjacent to RAPO-1-3, only a trace amount of Onjisaponin B was detected. To determine whether Onjisaponin B functioned as RAPO-1-3, we assessed Onjisaponin B for its Aβ reducing activity using the HEK293/APPswe cell line. Tenuifolin was compared in parallel as a core structure control. Indeed, Onjisaponin B exhibited comparable Aβ reducing activity with RAPO-1-3 with an IC50 of 10 μM, while Tenuifolin showed no effect (Fig 2D and 2E). Neither Onjisaponin B nor Tenuifolin showed obvious cytotoxicity during the treatment as revealed by the CellTiter-Glo test (Fig 2D and 2E). Then, we examined whether Onjisaponin B affected BACE1 and γ-secretase activities. Surprisingly, Onjisaponin B enhanced BACE1 activity in the ELISA-based BACE1 activity assay (Fig 2F). However, the western blots for the processing patterns of the physiological BACE1 substrates APP and PSGL1 were not altered upon Onjisaponin B treatment (Figs 2G and 3B). Meanwhile, γ-secretase activity was not changed upon Onjisaponin B treatment (Fig 2F). Furthermore, we examined the effects of these compounds on NotchΔE processing. We did not observe effects of Onjisaponin B on the processing of the γ-secretase substrate NotchΔE (Fig 2H). Thus, the function of Onjisaponin B resembles that of RAPO-1-3, reducing Aβ production without directly interfering with BACE1 or γ-secretase enzymatic activity. Moreover, unlike the γ-secretase modulator (GSM) ibuprofen or the GSI Compound E, Onjisaponin B did not affect the efficiency of PS1 internal FRET [28] (S4C Fig), suggesting that Onjisaponin B is neither a GSM nor a GSI. Next, we acutely administered RAPO-1-3 (0.15 g/kg/day) or Onjisaponin B (10 mg/kg/day) to C57BL/6 mice. Plasma was collected after 10 minutes, 30 minutes, 1 hour, 2 hours, 3 hours, 5 hours and 24 hours, and the concentration of Onjisaponin B in the plasma was detected. Plasma from the vehicle-treated mice were also collected and measured as background signal. Onjisaponin B was detectable with either RAPO-1-3 or Onjisaponin B treatment. The level of Onjisaponin B was approximately 4 times higher in the plasma of RAPO-1-3-treated mice than in that of the Onjisaponin B-treated group (Fig 2I).

### RAPO-1-3 and Onjisaponin B promote APP degradation

Studies show that interference with APP metabolism alters Aβ production [16, 18, 48–51]. Thus, we monitored the APP cleavage pattern on western blots. Treatment with BSI IV completely abolished the production of sAPPβ and C99 and led to the accumulation of mature APP and C83. GSI L685,458 treatment caused the accumulation of C99 and C83, while the GSM E2012 exerted little effect on any of the APP metabolic products (Fig 3A). Conversely, treatment with RAPO, RAPO-1 or RAPO-1-3 led to a reduction in the level of full-length mature APP, and accordingly the protein levels of the cleavage products (including sAPPα, C83, sAPPβ and C99), while the immature APP level remained unchanged (Fig 3A). Onjisaponin B treatment resulted in a similar APP processing pattern (Fig 3B) as that of RAPO-1-3. Nevertheless, neither BACE1 expression nor its maturation changed significantly upon Onjisaponin B treatment (S3A Fig). These data suggest that Onjisaponin B may not reduce the cellular Aβ level by blocking APP maturation. To test whether Onjisaponin B inhibited APP or secretase expression, we performed quantitative RT-PCR analysis. As shown in S3C Fig, we found no obvious differences among the mRNA levels of those proteins. Then, we tested whether it was the alteration of APP protein levels that led to the reduction of Aβ production. The *in vitro* APP processing in the presence of active BACE1 was monitored. HEK293/APPswe cell membrane fractions were extracted and incubated with the indicated compounds for 2 hours in BACE1-assay buffer. Treatment with BSI IV completely blocked sAPPβ generation and caused the accumulation of full-length mature APP and C99, while GSI L685,458 did not change the sAPPβ level but caused minor accumulation of APP and C99 (Fig 3C). However, both treatment with either Onjisaponin B or Tenuifolin reduced the level of sAPPβ while leaving the level of full-length APP unchanged (Fig 3C). Further, in the presence of Onjisaponin B together with BSI IV, the full-length mature APP accumulated to a level comparable to BSI IV alone, whereas the level of sAPPβ was decreased (Fig 3D). Co-treatment with L685,458 and Onjisaponin B showed no synergetic effect compared to L685,458 treatment alone (Fig 3D). Moreover, an *in vitro* γ-secretase activity analysis showed that the AICD production was not affected by Onjisaponin B treatment (S3B Fig). These data suggest that Onjisaponin B reduced Aβ production neither by the direct inhibition of secretase activities nor by modulating protein expression. The proteasome proteolytic pathway has also been shown to be involved in the degradation of full-length APP [13, 52, 53]. Thus, we treated HEK293/APPswe cells with BSI IV or L685,458 in the presence of the proteasome inhibitor MG132 and monitored the APP processing pattern. Consistent with previous reports [54], MG132 showed no obvious effect on the APP processing pattern in the presence of BACE1 or γ-secretase inhibitors (Fig 3E). Interestingly, the decreases in full-length mature APP and subsequently in C99 and C83 by Onjisaponin B were partially rescued in the presence of MG132 (Fig 3E). The Aβ levels of the cells treated with MG132 and Onjisaponin B were comparable to those of the cells treated with MG132 alone (Fig 3F). To rule out the possibility that the rescue by MG132 was due to its GSI-like activity, we tested another, more specific proteasome inhibitor, lactacystin, which has been reported to show no GSI-like activity even at 100 μM [55]. As shown in Fig 3F, the Aβ levels of the cells treated with Onjisaponin B in the presence of lactacystin were significantly higher than those of the cells treated with Onjisaponin B alone. Together, these data indicate that Onjisaponin B and the active fractions of RAPO reduce Aβ production by promoting APP degradation through the proteasome pathway.

Additionally, as we recently reported that blocking the PS1/BACE1 interaction could also contribute to reduced production of Aβ [19], we tested whether Onjisaponin B possessed a similar inhibitory activity. Co-immunoprecipitation analysis of PS1 and BACE1 was performed. As shown in S4A Fig, Onjisaponin B interfered with the interaction between PS1 and BACE1. Further, FRET [19] (S4B Fig) and Split-TEV assay [19, 56] (S4D Fig) also showed that Onjisaponin B reduced the interaction between PS1 and BACE1. These data indicate that Onjisaponin B may possess multiple functions to reduce Aβ production.

### Onjisaponin B ameliorates cognitive impairments in APP/PS1 mice

**Fig 4:**
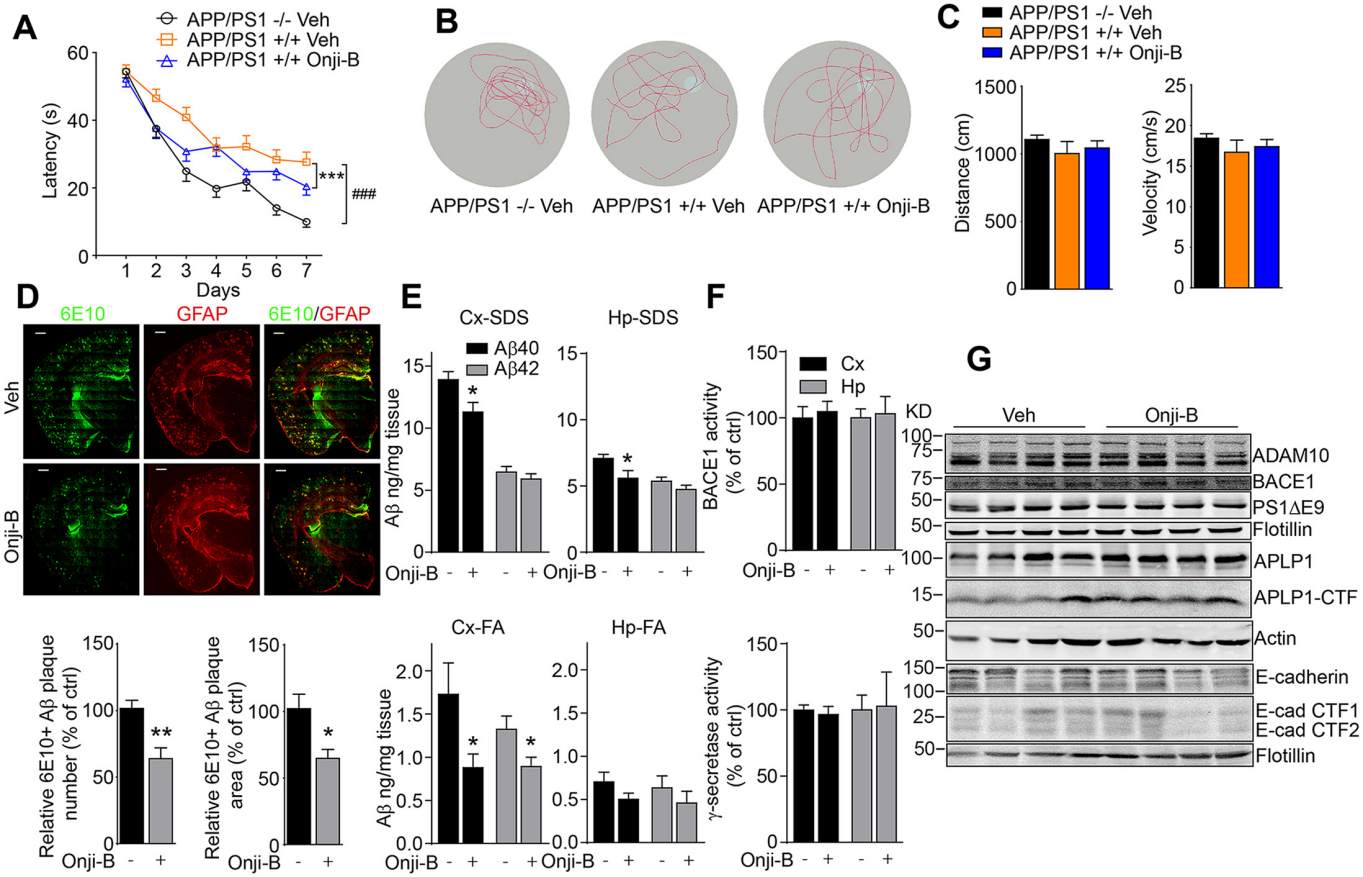
Onjisaponin B ameliorates cognitive impairments in APP/PS1 mice. (A) Onjisaponin B significantly attenuates spatial memory deficits of AD mice in the Morris Water Maze test. (B) Representative tracks of each group of mice in the probe trial test at day 8. (C) The swimming distance and velocity of mice. (D) Representative images of Aβ plaques in APP/PS1 mice immunostained with the Aβ antibody 6E10 in coronal mouse brain cryo-sections (n = 6 per group). The number and area of Aβ plaques, which are immunoreactive to 6E10, were quantified from entire brain sections using Image-Pro Plus 5.1 software (Media Cybernetics). Ten to fifteen coronal sections per mouse were analyzed. (E) SDS-soluble and FA-soluble Aβ40 and Aβ42 levels in mouse hippocampus and cortex measured by ELISA. (F) Mouse hippocampal and cortical BACE1 and γ-secretase activities and (G) Western blot analysis of cortical protein extracts with indicated antibodies. Data are presented as the mean ± s.e.m. * *p* < 0.05, ** *p* < 0.01 and *** *p* < 0.001. Two-way ANOVA with Bonferroni's multiple comparison test (A), one-way ANOVA with Bonferroni's multiple comparison test (C) and two-tailed *t*-test (D, E and F).

Because Onjisaponin B demonstrated the functions of RAPO-1-3 and reduced Aβ production at the cellular level, we further tested whether Onjisaponin B was effective *in vivo*. We chronically administered Onjisaponin B to APP/PS1 mice. The drug administration began prophylactically at 4 months of age. Gender-and age-matched APP/PS1 transgenic mice and their WT littermates were grouped and treated with Onjisaponin B (10 mg/kg/day) or Vehicle (50% PEG400 in distilled water) by oral gavage. During Onjisaponin B administration, animals’ body weights were recorded and no significant differences were observed among the groups (S5A Fig), showing that there were no obvious toxic effects in mice. After three months of drug administration, the mice were subjected to the Morris Water Maze analysis to evaluate their spatial learning and reference memory ability. The swimming distance and velocity remained at comparable levels among different groups of mice (Fig 4C), implying that treatment with Onjisaponin B does not alter mouse locomotor activity. As shown in Fig 4A, consistent with previous results, APP/PS1 mice exhibited significantly impaired learning and memory ability in the hidden platform phase (Veh APP/PS1 -/- vs. Veh APP/PS1 +/+, *p* < 0.0001). This spatial learning and memory deficit in APP/PS1 mice was ameliorated by chronic administration of Onjisaponin B (Onji-B APP/PS1 +/+ vs. Veh APP/PS1 +/+, *p* = 0.0002). During the probe trial on day 8, the mice treated with Onjisaponin B were faster in reaching the position of the platform (S5B Fig), spent more time in the target quadrant (S5C Fig), and crossed slightly more frequently within the platform area (Fig 4B) than the vehicle-treated APP/PS1 +/+ ones. These data indicate that Onjisaponin B effectively ameliorates the spatial learning and reference memory deficiency of APP/PS1 transgenic mice.

Along with the AD-like symptoms, APP/PS1 mice develop cerebral amyloidosis [57, 58]. Hence, a histological analysis was performed to assess their AD-like pathology. Brains of mice treated with Onjisaponin B exhibited significantly lower numbers of 6E10-positive Aβ plaque and reduced plaque area compared to those of the vehicle-treated ones (Fig 4D and S5D Fig). SDS-soluble and FA-soluble Aβ40 and Aβ42 in mouse cortex and hippocampus were also monitored by ELISA. As shown in Fig 4E, SDS-soluble Aβ40 and Aβ42 levels were moderately reduced in cortical and hippocampal extracts of the Onjisaponin B-treated group, while the levels of FA-soluble Aβ40 and Aβ42 were significantly reduced. Further assays of secretase activity revealed that none of the activities (Fig 4F) or the expression levels (Fig 4G) of BACE1 or γ-secretase in the brain extracts of the Onjisaponin B-treated mice altered. Moreover, the processing of other secretase substrates, i.e., APLP1 and E-cadherin, were also similar in mouse brain samples (Fig 4H). These data indicate that Onjisaponin B possesses *Radix Polygalae* activity and may ameliorate Aβ pathology *in vivo*.

## Discussion

Drugs designed for Alzheimer’s disease currently mostly focus on reducing Aβ levels, which appears to be very challenging [59]. Direct inhibitors of secretases effectively inhibited Aβ production *in vitro* but showed potential problems during clinical trials. Volunteers endured many adverse effects including headache, skin cancer, and liver failure [60–62]. Researchers also tested GSMs that moderately regulate the Aβ species produced, for which the clinical trials are still in progress [63]. Unlike these secretase inhibitors and modulators, RAPO, its active fraction RAPO-1-3 and the functional representative constituent Onjisaponin B reduced the Aβ level without interfering with the enzymatic activities of secretases. There is also a certain amount of Onjisaponin B in RAPO-3-2 based on the UPLC-Q-TOF/MS analysis of RAPO-3-2 (S2H Fig). However, there are differences in the peak patterns of RAPO-1-3 and RAPO-3-2 (S2B and S2G Fig). The cytotoxicity introduced by RAPO-3-2 treatment may result from other constituents in the fraction, which requires further investigation, and prompted us to abandon testing the *in vivo* efficacy of RAPO-3-2. We are continuing to attempt to separate the Aβ-reducing activity and the cytotoxicity of RAPO-3-2.

According to a previous reports, saponins were enriched in ethanol or methanol fractions [64] and the BPI chromatogram of RAPO-3-2 supports this conclusion (S2G and S2H Fig). Moreover, saponins were also presented in RAPO-1 and RAPO-1-3 (S2A–S2D Fig). Using a two-step column chromatography separation method, we identified the major constituents in RAPO-1-3. In the UPLC-Q-TOF/MS chromatogram (S2A and S2B Fig), the representative acyl saponins peaks were observed at UV 310 nm. The total 10 related components were characterized as acyl saponins from RAPO-1-3 based on accurate mass measurement (S2H Fig and S1 Table). Those enriched acyl saponins possess highly similar structures, which makes it remarkably difficult to discriminate each compound from the others and certainly made it difficult to obtain an accurate plasma concentration of Onjisaponin B after drug administration. Due to technical problems, it is not clear why the plasma concentration of Onjisaponin B after RAPO-1-3 treatment was 10-folds higher than that after Onjisaponin B treatment at the 30-minute time point alone. It is possible that other compounds in RAPO-1-3 may enhance the absorption of Onjisaponin B and that other many saponins such as Onjisaponin R, S and Ng with similar polarity to Onjisaponin B may interfere with the peak area of Onjisaponin B. In addition, Onjisaponin B may have a relatively short half-life *in vivo*, and there might be other compounds in RAPO-1-3 that may be converted into Onjisaponin B and help to maintain the plasma drug concentration *in vivo*. All those possibilities must be further investigated. The higher level of Onjisaponin B *in vivo* after acute administration with RAPO-1-3 than after Onjisaponin B treatment alone might also explain why RAPO-1-3-treated AD mice performed better in the Morris Water Maze than did the Onjisaponin B-treated group.

There is evidence that proteostasis is disturbed in the brains of AD carriers [65]. As an important part of protein homeostasis, protein degradation plays a vital role in cleaning misfolded proteins, aggregated proteins or proteins no longer in use [66, 67]. This process can be accomplished directly, by the proteasome, or through other mechanisms such as autophagy [67]. Further mechanistic studies show that, together with our finding that Onjisaponin B reduces the Aβ level by promoting APP degradation, it is possible that the autophagy-related Atg7-dependent AMPK/mTOR pathway may also be involved in Aβ clearance, as in previous reports [68, 69].

To introduce another possible target for the modulation of AD, the interaction between BACE1 and γ-secretase and their chemical blockers have recently been reported by our lab [19]. Here, we found that Onjisaponin B also functioned as a mild inhibitor of the PS1/BACE1 interaction. Interestingly, Onjisaponin B interferes with the PS1/BACE1 interaction in an immunoprecipitation assay, reduces their FRET efficiency and dose-dependently reduces PS1-NTF/BACE1 interaction signal in a Split-TEV reporter assay.

RAPO is traditionally believed to harbor nootropic and tranquilizing efficacy, and it has therefore been used historically in China as a medical herb for memory deficits and insomnia [20]. Studies showed that RAPO produced a neuro-protective effect in a Parkinson’s disease model [70], improved learning and memory ability in Alzheimer’s disease animal models [71], and exhibited anti-depressant [72] and anti-inflammatory [73] activity. The underlying mechanisms include enhancing choline acetyl-transferase activity and nerve growth factor secretion [74], promoting BDNF and TrkB expression [75], and activating the NF-κB pathway [76], etc. Here, we report that Onjisaponin B from RAPO reduces Aβ production by promoting APP degradation. It is fascinating to find that such one natural product could exhibit multiple functions, and these results support the new paradigm of Multi-Target-Directed Ligands (MTDLs) [77–79].

## Supporting Information

S1 Document The individual identification report of the components in RAPO-1-3. (PDF)

S1 Fig Total secreted Aβ in HEK293/APPswe culture medium. Cells were treated with 0.1, 0.3 and 1 mg/ml RAPO or its fractions for 8 hours as indicated (A), and cell viability was monitored in parallel (B). Data are presented as the mean ± s.e.m. * *p* < 0.05, ** *p* < 0.01 and *** *p* < 0.001. One-way ANOVA with Bonferroni's multiple comparison test (A, B).
(TIF)

S2 Fig RAPO-1-3 and RAPO-3-2 characterization. (A) UPLC chromatogram of RAPO-1-3 at 310 nm. (B) BPI chromatogram of RAPO-1-3 in negative ion mode. (C) Chemical structures of saponins identified in RAPO-1-3. (D) EIC chromatogram of Onjisaponin B in RAPO-1-3. (E) EIC chromatogram of Onjisaponin B at 500 μg/ml. (F) EIC chromatogram of Onjisaponin B at 100 μg/ml. (G) BPI chromatogram of RAPO-3-2 in negative mode. (H) EIC chromatogram of Onjisaponin B in RAPO-3-2.
(TIF)

S3 Fig Onjisaponin B does not affect BACE1 maturation or γ-secretase activity or the secretase expression level *in vitro*. (A) Onjisaponin B (10 μM) does not alter the level of mature BACE1. (B) No significant alteration in the C99 processing pattern in the presence of 10 μM Onjisaponin B. (C) mRNA levels of APP, BACE and γ-secretase components upon 10 μM Onjisaponin B treatment. Data are presented as the mean ± s.e.m. * *p* < 0.05, ** *p* < 0.01 and *** *p* < 0.001. One-way ANOVA with Bonferroni's multiple comparison test (C).
(TIF)

S4 Fig Onjisaponin B interferes with PS1/BACE1 interaction. (A) Onjisaponin B reduces PS1/BACE1 interaction. (B) Onjisaponin B reduces FRET efficiency of CFP-PS1/BACE1-YFP. (C) Onjisaponin B does not interfere with PS1 internal FRET efficiency. (D) Onjisaponin B dose-dependently reduces PS1-NTF/BACE1 interaction in Split-TEV luminescent reporter assay. Data are presented as the mean ± s.e.m. * *p* < 0.05, ** *p* < 0.01 and *** *p* < 0.001. One-way ANOVA with Bonferroni's multiple comparison test (B, C) and one-way ANOVA with the Holm-Sidak multiple comparison test (D).
(TIF)

S5 Fig Mouse body weight during compound administration, probe trial data at day 8 and representative images of brain slice immunostained with Aβ and astrocyte. (A) Monthly body weight. (B) Latency to platform in the probe trial. (C) Time spent in the target quadrant for each group. Data are presented as the mean ± s.e.m. * *p* < 0.05, ** *p* < 0.01 and *** *p* < 0.001. Two-way ANOVA with Bonferroni's multiple comparison test (A, C), one-way ANOVA with Bonferroni's multiple comparison test (B). (D) More representative images of amyloid-β plaques in APP/PS1 mice immunostained with Aβ antibody 6E10 in coronal mouse brain cryo-sections.
(TIF)

S1 Protocol Supplementary material and methods. (DOCX)

S1 Table Main compounds identified in RAPO-1-3 by UPLC-ESI-Q-TOF/MS. (PDF)

S2 Table The detailed information of the main peak areas in RAPO-1-3. (PDF)
