# Supplementary material for: Traditional Chinese Nootropic Medicine Radix Polygalae and Its Active Constituent Onjisaponin B Reduce β-Amyloid Production and Improve Cognitive Impairments
Source: PLoS One. 2016 Mar 8;11(3):e0151147. doi: 10.1371/journal.pone.0151147 (PMC4782990; doi:10.1371/journal.pone.0151147)
Supplement: S1 Document — (PDF) [file pone.0151147.s001.pdf]

Single Mass Analysis

Tolerance = 5.0 mDa / DBE: min = -1.5, max = 50.0
Element prediction: Off
Number of isotope peaks used for i-FIT = 3

Monoisotopic Mass, Even Electron Ions
1207 formula(e) evaluated with 3 results within limits (up to 20 best isotopic matches for each mass)
Elements Used:
C: 0-500 H: 0-1000 O: 0-200

RAPO-1-3
20160104-10 1418 (11.356) Cm (1399:1418) 1: TOF MS ES-
5.98e+003

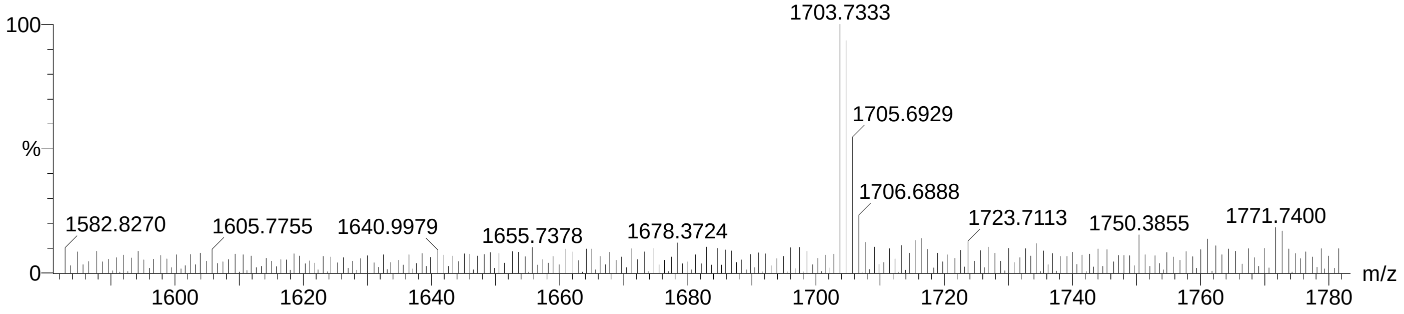

Minimum:				-1.5				
Maximum:		5.0	10.0	50.0				

Mass	Calc. Mass	mDa	PPM	DBE	i-FIT	Norm	Conf(%)	Formula
1703.7333	1703.7328	0.5	0.3	21.5	27.6	0.199	81.97	C80 H119 O39
	1703.7364	-3.1	-1.8	43.5	29.2	1.751	17.36	C98 H111 O26
	1703.7293	4.0	2.3	-0.5	32.4	5.010	0.67	C62 H127 O52

Single Mass Analysis

Tolerance = 5.0 mDa / DBE: min = -1.5, max = 50.0
Element prediction: Off
Number of isotope peaks used for i-FIT = 3

Monoisotopic Mass, Even Electron Ions
1017 formula(e) evaluated with 2 results within limits (up to 20 best isotopic matches for each mass)
Elements Used:
C: 0-500 H: 0-130 O: 0-200

RAPO-1-3
20160104-10 1457 (11.670) 1: TOF MS ES-
1.57e+002

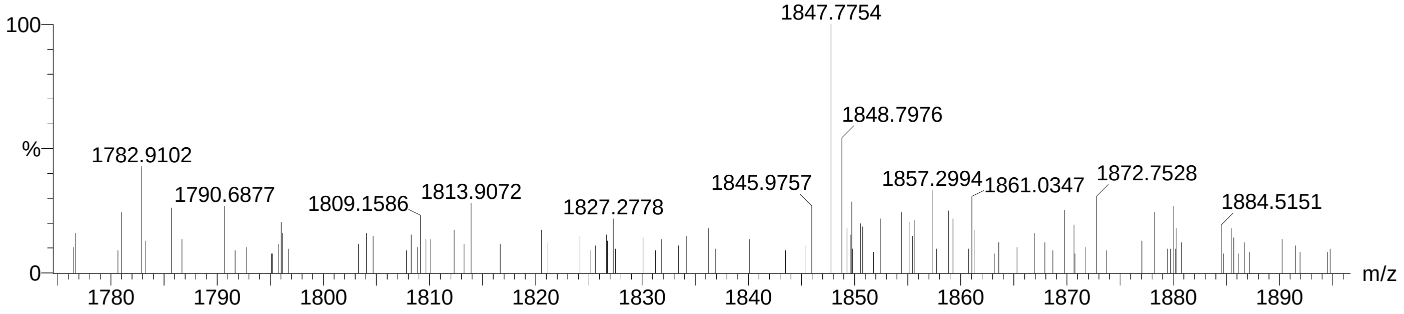

Minimum:				-1.5				
Maximum:		5.0	10.0	50.0				

Mass	Calc. Mass	mDa	PPM	DBE	i-FIT	Norm	Conf(%)	Formula
1847.7754	1847.7751	0.3	0.2	23.5	37.1	0.272	76.17	C86 H127 O43
	1847.7786	-3.2	-1.7	45.5	38.2	1.434	23.83	C104 H119 O30

Elemental Composition Report

Single Mass Analysis

Tolerance = 5.0 mDa / DBE: min = -1.5, max = 50.0
Element prediction: Off
Number of isotope peaks used for i-FIT = 3

Monoisotopic Mass, Even Electron Ions
899 formula(e) evaluated with 4 results within limits (up to 20 best isotopic matches for each mass)
Elements Used:
C: 0-500 H: 0-1000 O: 0-200

RAPO-1-3
20160104-10 1483 (11.876) Cm (1476:1486) 1: TOF MS ES- 4.05e+003

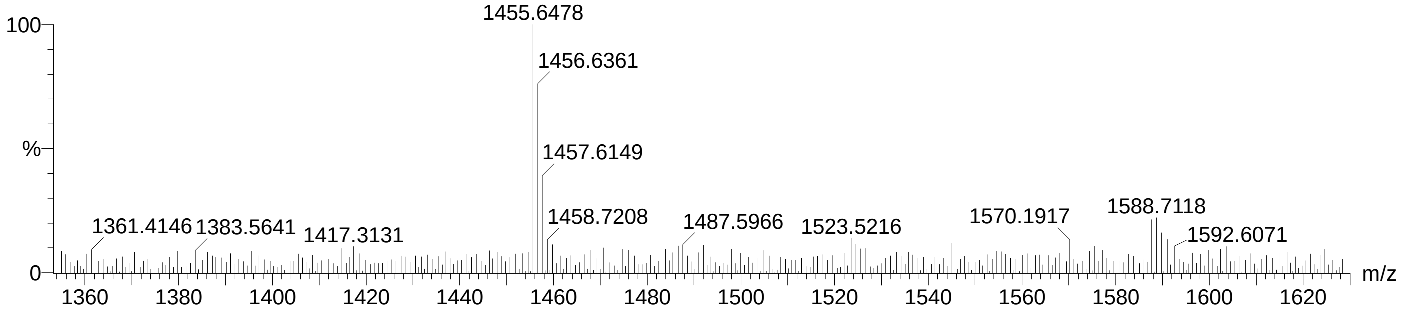

Minimum:				-1.5				
Maximum:		5.0	10.0	50.0				
Mass	Calc. Mass	mDa	PPM	DBE	i-FIT	Norm	Conf(%)	Formula
1455.6478	1455.6432	4.6	3.2	19.5	21.6	0.054	94.73	C70 H103 O32
	1455.6491	-1.3	-0.9	10.5	24.9	3.376	3.42	C63 H107 O37
	1455.6526	-4.8	-3.3	32.5	25.7	4.186	1.52	C81 H99 O24
	1455.6468	1.0	0.7	41.5	27.2	5.705	0.33	C88 H95 O19

Elemental Composition Report

Single Mass Analysis

Tolerance = 5.0 mDa / DBE: min = -1.5, max = 50.0
Element prediction: Off
Number of isotope peaks used for i-FIT = 3

Monoisotopic Mass, Even Electron Ions
1057 formula(e) evaluated with 3 results within limits (up to 20 best isotopic matches for each mass)
Elements Used:
C: 0-500 H: 0-1000 O: 0-200

RAPO-1-3
20160104-10 1568 (12.556) Cm (1560:1568)
1: TOF MS ES-
4.11e+003

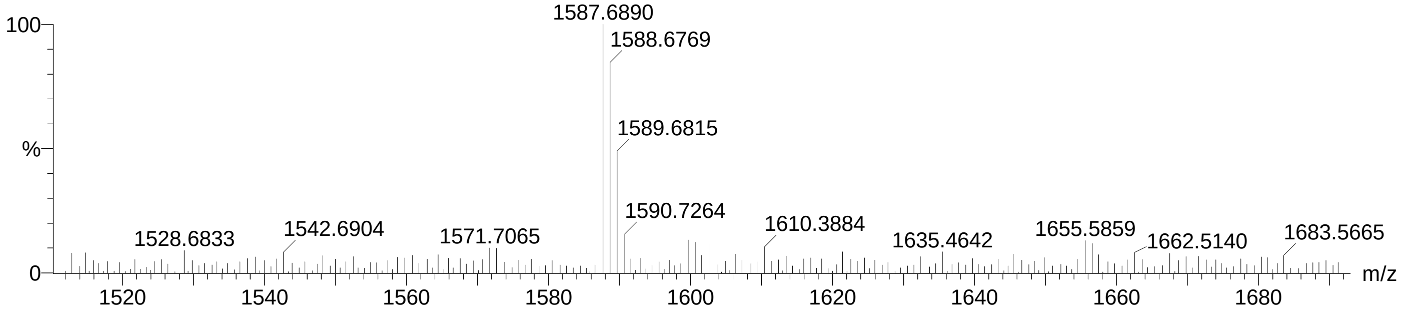

Minimum:				-1.5				
Maximum:		5.0	10.0	50.0				

Mass	Calc. Mass	mDa	PPM	DBE	i-FIT	Norm	Conf(%)	Formula
1587.6890	1587.6855	3.5	2.2	20.5	21.3	0.156	85.56	C75 H111 O36
	1587.6890	0.0	0.0	42.5	23.5	2.354	9.50	C93 H103 O23
	1587.6914	-2.4	-1.5	11.5	24.1	3.008	4.94	C68 H115 O41

Single Mass Analysis

Tolerance = 5.0 mDa / DBE: min = -1.5, max = 50.0
Element prediction: Off
Number of isotope peaks used for i-FIT = 3

Monoisotopic Mass, Even Electron Ions
218 formula(e) evaluated with 1 results within limits (up to 20 best isotopic matches for each mass)
Elements Used:
C: 65-85 H: 0-130 O: 0-70
RAPO-1-3
20160104-10 1621 (12.986) Cm (1614:1637) 1: TOF MS ES- 7.82e+003

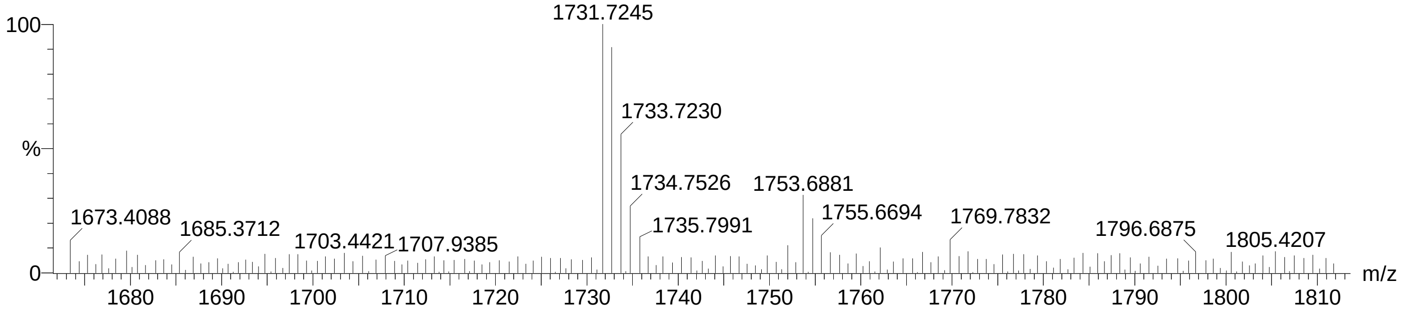

Minimum:				-1.5				
Maximum:		5.0	10.0	50.0				
Mass	Calc. Mass	mDa	PPM	DBE	i-FIT	Norm	Conf(%)	Formula
1731.7245	1731.7278	-3.3	-1.9	22.5	21.3	n/a	n/a	C81 H119 O40

Single Mass Analysis

Tolerance = 5.0 mDa / DBE: min = -1.5, max = 50.0
Element prediction: Off
Number of isotope peaks used for i-FIT = 3

Monoisotopic Mass, Even Electron Ions
218 formula(e) evaluated with 2 results within limits (up to 20 best isotopic matches for each mass)
Elements Used:
C: 65-85 H: 0-130 O: 0-70
RAPO-1-3
20160104-10 1869 (14.964) Cm (1862:1872)
1: TOF MS ES-
8.24e+003

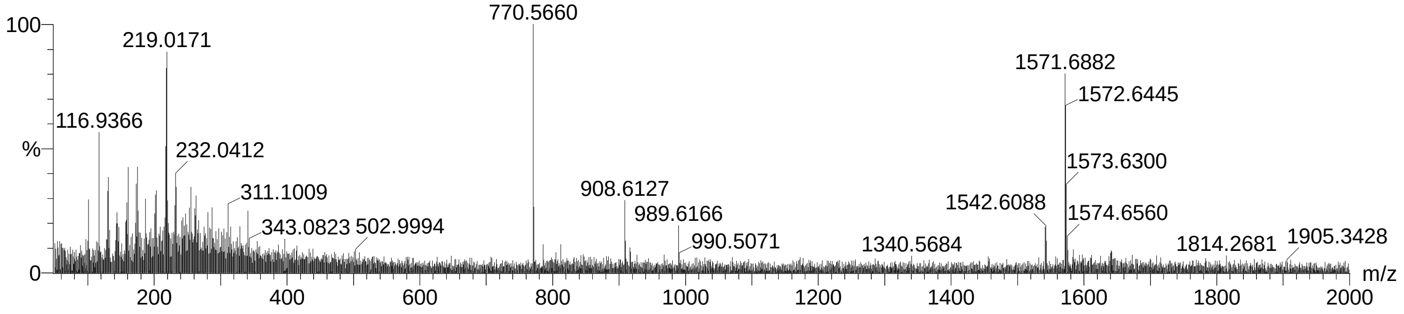

Minimum:				-1.5				
Maximum:		5.0	10.0	50.0				
Mass	Calc. Mass	mDa	PPM	DBE	i-FIT	Norm	Conf(%)	Formula
1571.6882	1571.6906	-2.4	-1.5	20.5	24.9	0.149	86.17	C75 H111 O35
	1571.6847	3.5	2.2	29.5	26.7	1.978	13.83	C82 H107 O30

Single Mass Analysis

Tolerance = 5.0 mDa / DBE: min = -1.5, max = 50.0
Element prediction: Off
Number of isotope peaks used for i-FIT = 3

Monoisotopic Mass, Even Electron Ions
217 formula(e) evaluated with 2 results within limits (up to 20 best isotopic matches for each mass)
Elements Used:
C: 65-85 H: 0-130 O: 0-70
RAPO-1-3
20160104-10 1971 (15.783) Cm (1917:1982) 1: TOF MS ES- 8.51e+003

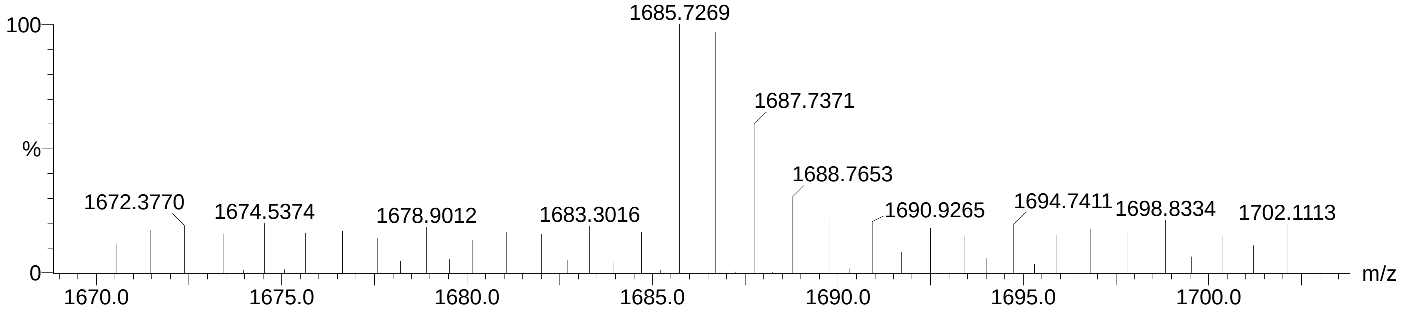

Minimum:				-1.5				
Maximum:		5.0	10.0	50.0				

Mass	Calc. Mass	mDa	PPM	DBE	i-FIT	Norm	Conf(%)	Formula
1685.7269	1685.7223	4.6	2.7	22.5	33.8	0.149	86.20	C80 H117 O38
	1685.7282	-1.3	-0.8	13.5	35.6	1.980	13.80	C73 H121 O43

Single Mass Analysis

Tolerance = 5.0 mDa / DBE: min = -1.5, max = 50.0
Element prediction: Off
Number of isotope peaks used for i-FIT = 3

Monoisotopic Mass, Even Electron Ions
217 formula(e) evaluated with 2 results within limits (up to 20 best isotopic matches for each mass)
Elements Used:
C: 65-85 H: 0-130 O: 0-70

RAPO-1-3
20160104-10 2059 (16.486) Cm (2059:2081) 1: TOF MS ES-
2.18e+004

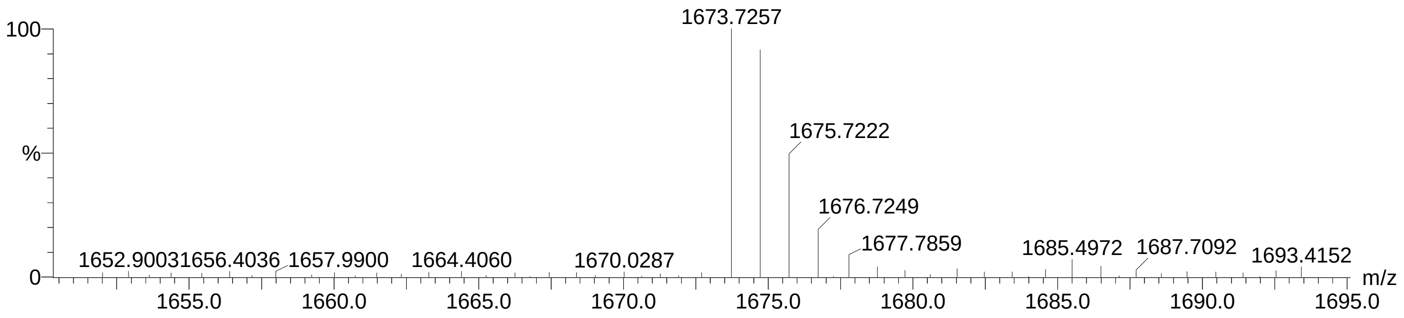

Minimum:				-1.5				
Maximum:		5.0	10.0	50.0				

Mass	Calc. Mass	mDa	PPM	DBE	i-FIT	Norm	Conf(%)	Formula
1673.7257	1673.7223	3.4	2.0	21.5	20.8	0.006	99.43	C79 H117 O38
	1673.7282	-2.5	-1.5	12.5	26.0	5.167	0.57	C72 H121 O43

Single Mass Analysis

Tolerance = 5.0 mDa / DBE: min = -1.5, max = 50.0
Element prediction: Off
Number of isotope peaks used for i-FIT = 3

Monoisotopic Mass, Even Electron Ions
217 formula(e) evaluated with 1 results within limits (up to 20 best isotopic matches for each mass)
Elements Used:
C: 65-85 H: 0-130 O: 0-70
RAPO-1-3
20160104-10 2167 (17.350) 1: TOF MS ES-
3.04e+002

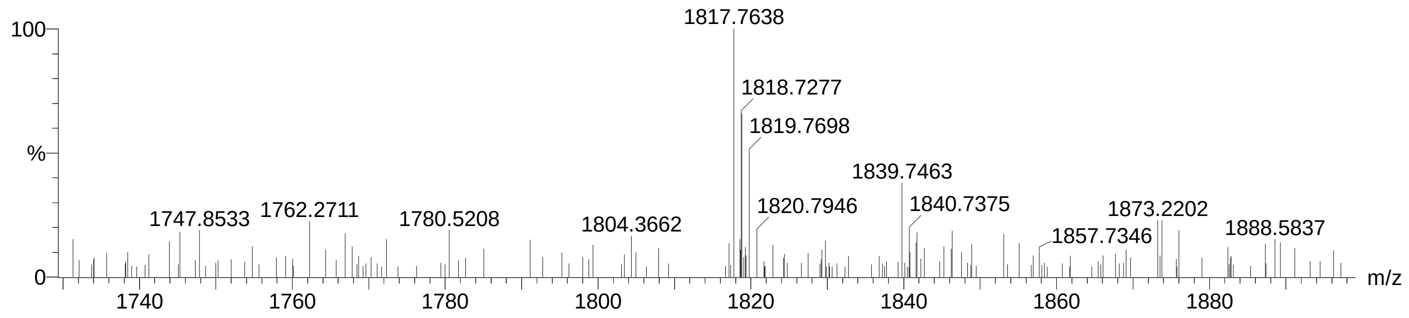

Minimum:				-1.5				
Maximum:		5.0	10.0	50.0				
Mass	Calc. Mass	mDa	PPM	DBE	i-FIT	Norm	Conf(%)	Formula
1817.7638	1817.7645	-0.7	-0.4	23.5	64.7	n/a	n/a	C85 H125 O42

Single Mass Analysis

Tolerance = 5.0 mDa / DBE: min = -1.5, max = 50.0
Element prediction: Off
Number of isotope peaks used for i-FIT = 3

Monoisotopic Mass, Even Electron Ions
217 formula(e) evaluated with 1 results within limits (up to 20 best isotopic matches for each mass)
Elements Used:
C: 65-85 H: 0-130 O: 0-70

RAPO-1-3
20160104-10 2376 (19.025) 1: TOF MS ES-
6.05e+002

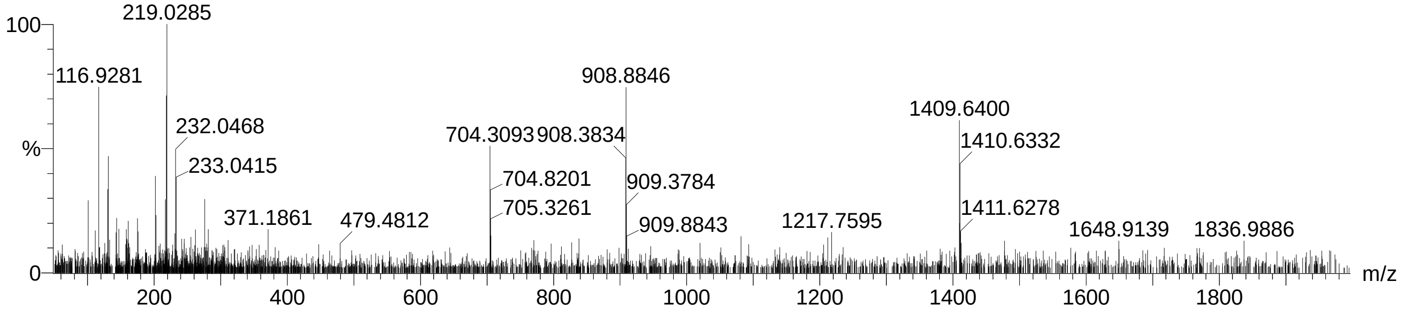

Minimum:				-1.5				
Maximum:		5.0	10.0	50.0				

Mass	Calc. Mass	mDa	PPM	DBE	i-FIT	Norm	Conf(%)	Formula
1409.6400	1409.6378	2.2	1.6	19.5	51.6	n/a	n/a	C69 H101 O30
