# Supplementary figures and images for: Traditional Chinese Nootropic Medicine Radix Polygalae and Its Active Constituent Onjisaponin B Reduce β-Amyloid Production and Improve Cognitive Impairments
Source: PLoS One. 2016 Mar 8;11(3):e0151147. doi: 10.1371/journal.pone.0151147 (PMC4782990; doi:10.1371/journal.pone.0151147)

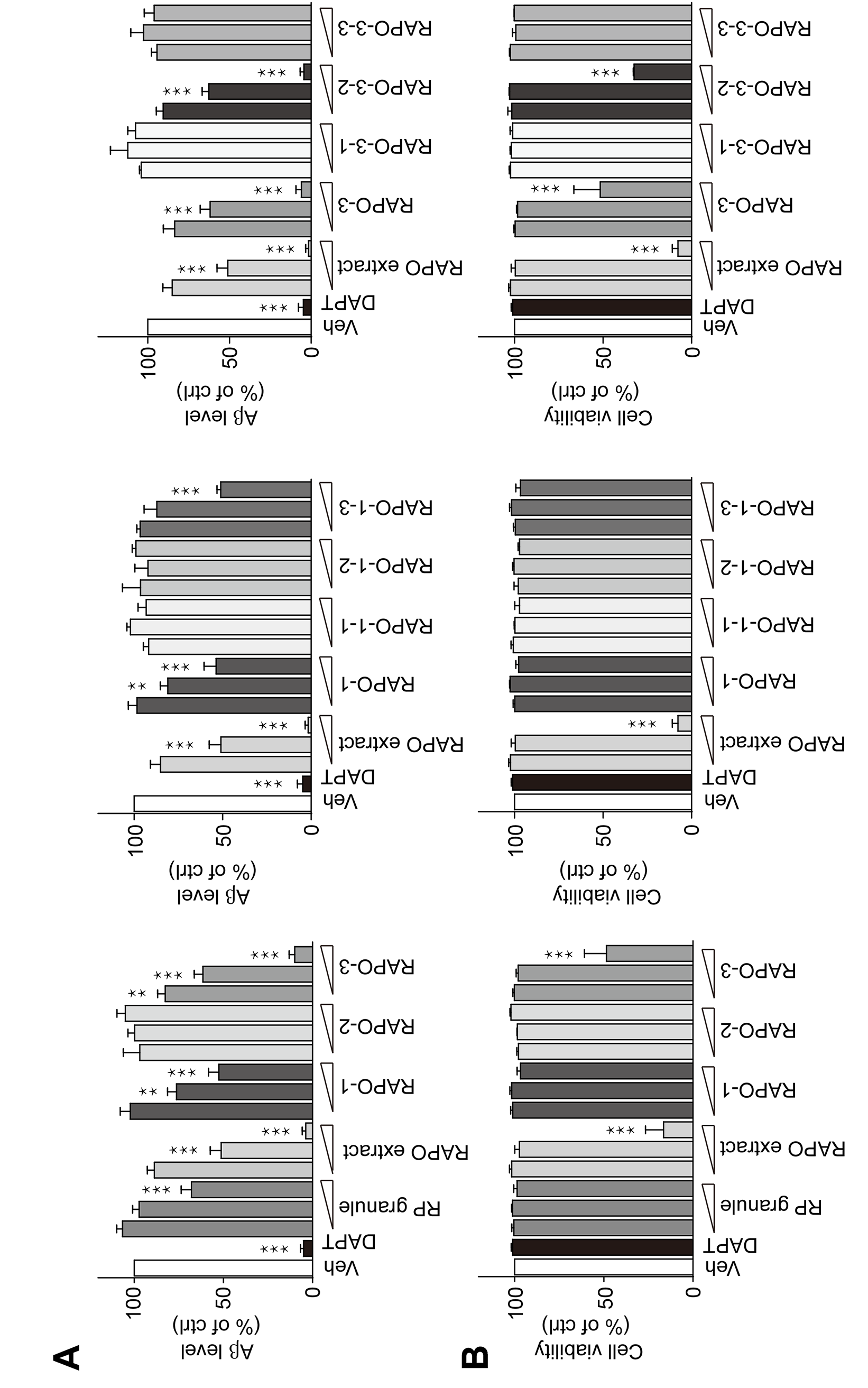

Supplement: S1 Fig — Cells were treated with 0.1, 0.3 and 1 mg/ml RAPO or its fractions for 8 hours as indicated (A), and cell viability was monitored in parallel (B). Data are presented as the mean ± s.e.m. * p < 0.05, ** p < 0.01 and *** p < 0.001. One-way ANOVA with Bonferroni's multiple comparison test (A, B). (TIF) [file pone.0151147.s002.tif]

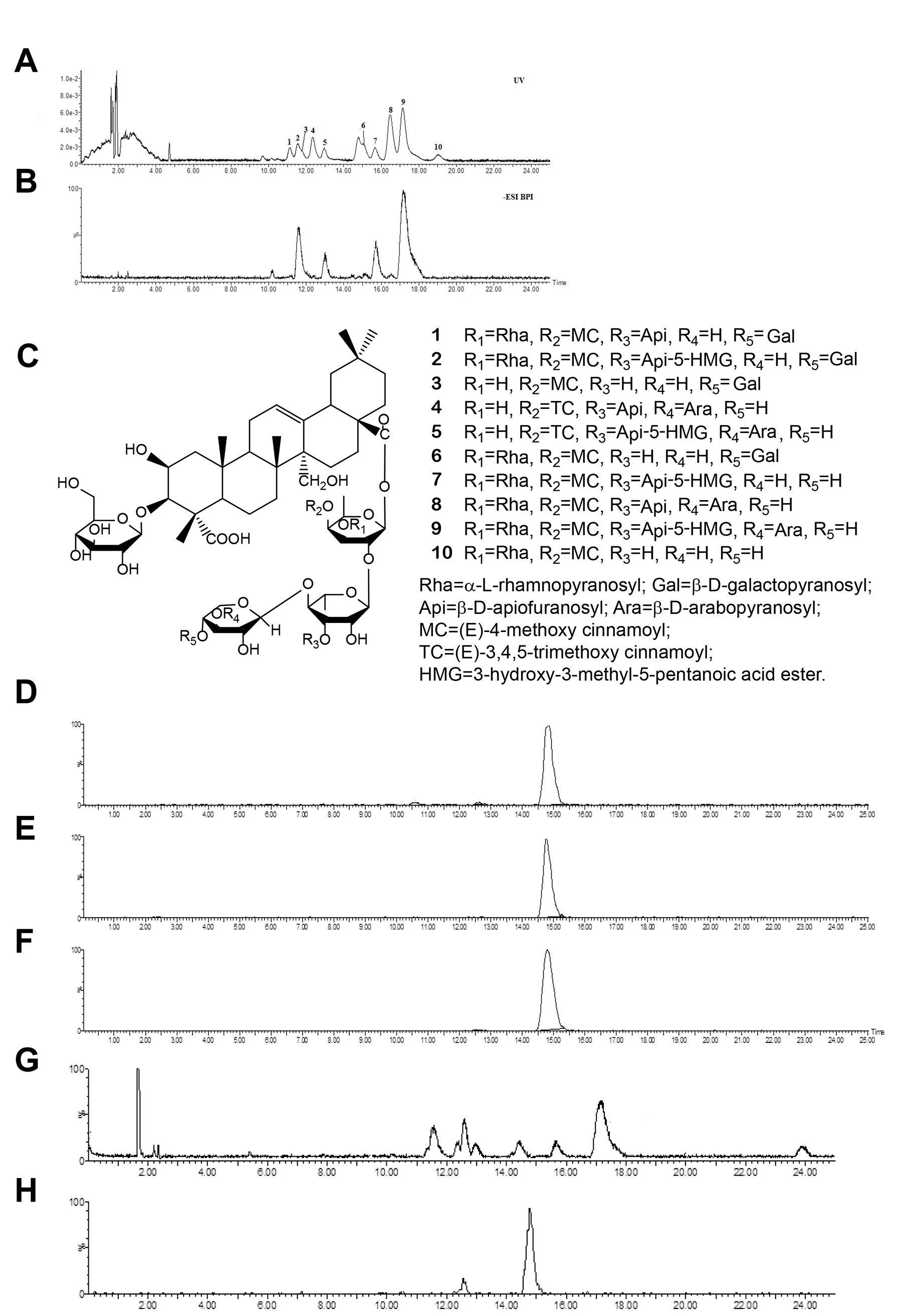

Supplement: S2 Fig — (A) UPLC chromatogram of RAPO-1-3 at 310 nm. (B) BPI chromatogram of RAPO-1-3 in negative ion mode. (C) Chemical structures of saponins identified in RAPO-1-3. (D) EIC chromatogram of Onjisaponin B in RAPO-1-3. (E) EIC chromatogram of Onjisaponin B at 500 μg/ml. (F) EIC chromatogram of Onjisaponin B at 100 μg/ml. (G) BPI chromatogram of RAPO-3-2 in negative mode. (H) EIC chromatogram of Onjisaponin B in RAPO-3-2. (TIF) [file pone.0151147.s003.tif]

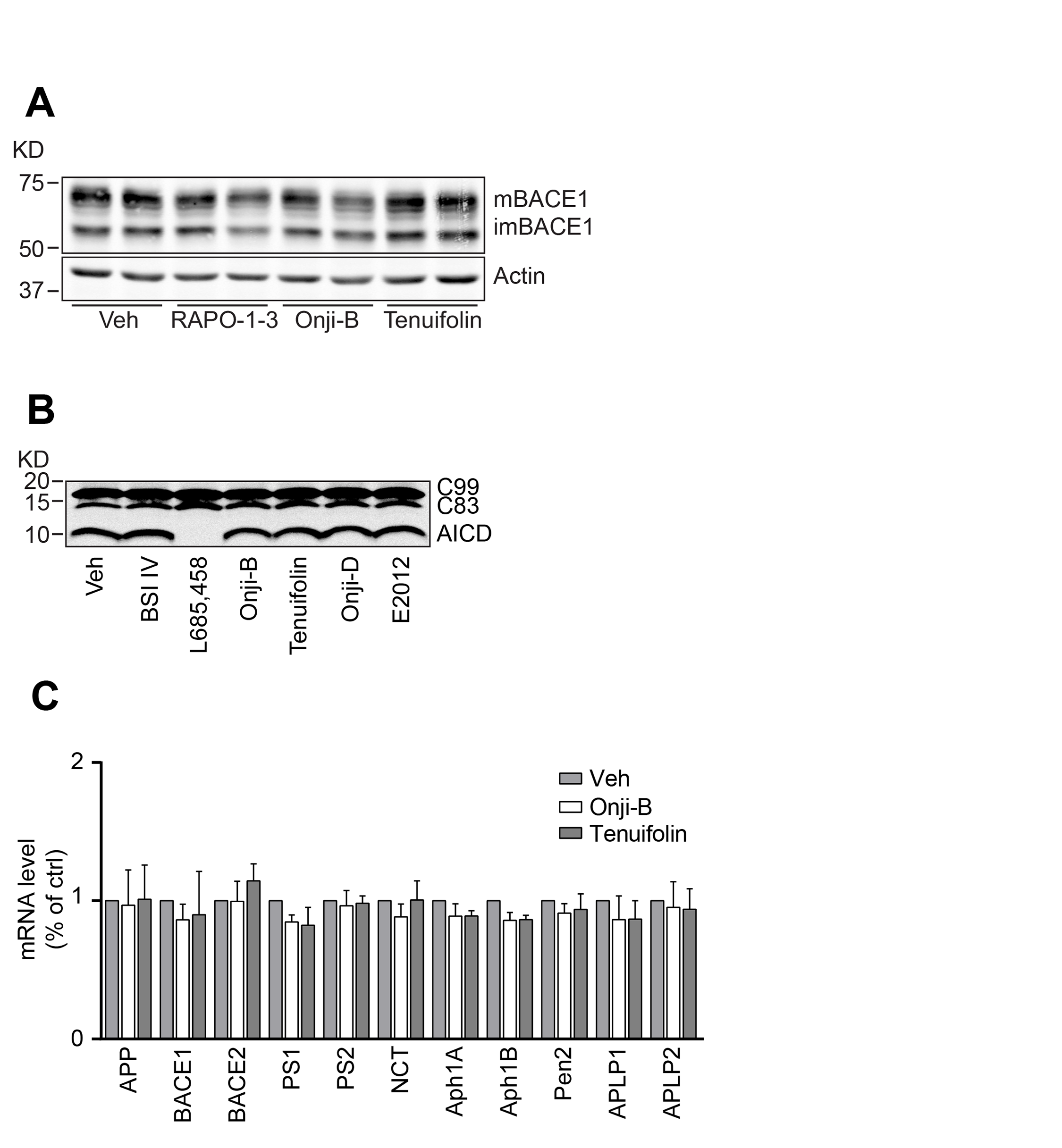

Supplement: S3 Fig — (A) Onjisaponin B (10 μM) does not alter the level of mature BACE1. (B) No significant alteration in the C99 processing pattern in the presence of 10 μM Onjisaponin B. (C) mRNA levels of APP, BACE and γ-secretase components upon 10 μM Onjisaponin B treatment. Data are presented as the mean ± s.e.m. * p < 0.05, ** p < 0.01 and *** p < 0.001. One-way ANOVA with Bonferroni's multiple comparison test (C). (TIF) [file pone.0151147.s004.tif]

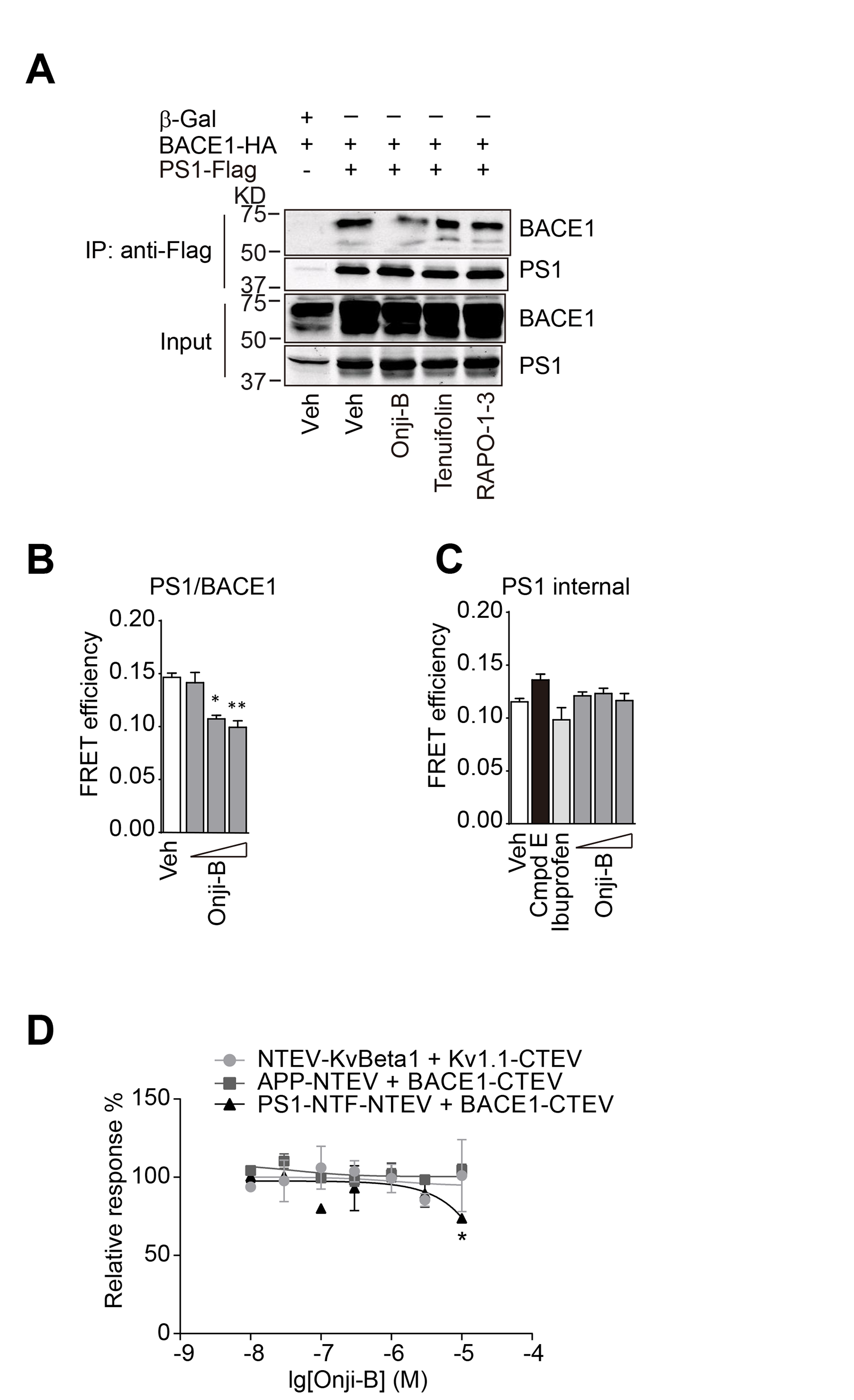

Supplement: S4 Fig — (A) Onjisaponin B reduces PS1/BACE1 interaction. (B) Onjisaponin B reduces FRET efficiency of CFP-PS1/BACE1-YFP. (C) Onjisaponin B does not interfere with PS1 internal FRET efficiency. (D) Onjisaponin B dose-dependently reduces PS1-NTF/BACE1 interaction in Split-TEV luminescent reporter assay. Data are presented as the mean ± s.e.m. * p < 0.05, ** p < 0.01 and *** p < 0.001. One-way ANOVA with Bonferroni's multiple comparison test (B, C) and one-way ANOVA with the Holm-Sidak multiple comparison test (D). (TIF) [file pone.0151147.s005.tif]

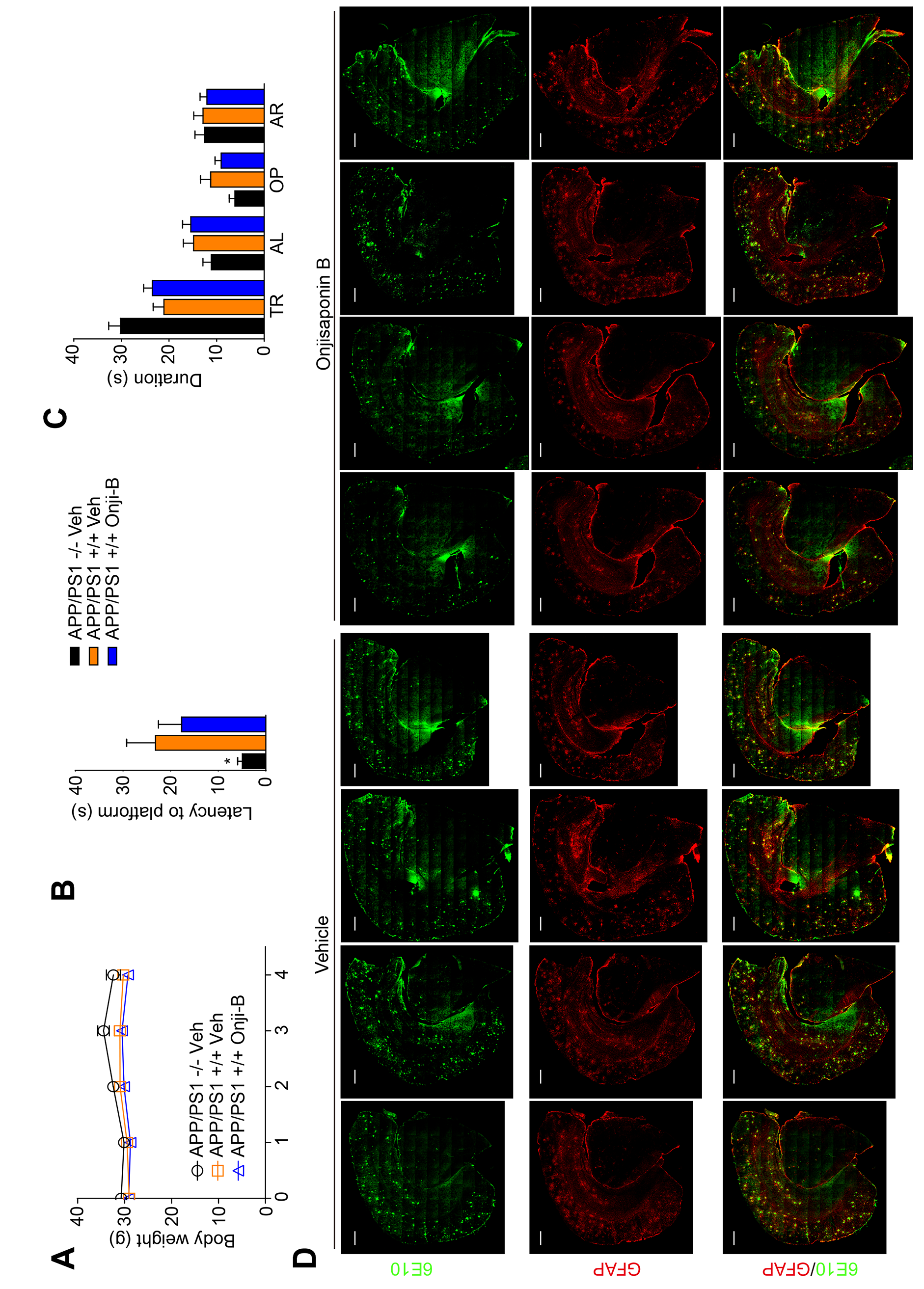

Supplement: S5 Fig — (A) Monthly body weight. (B) Latency to platform in the probe trial. (C) Time spent in the target quadrant for each group. Data are presented as the mean ± s.e.m. * p < 0.05, ** p < 0.01 and *** p < 0.001. Two-way ANOVA with Bonferroni's multiple comparison test (A, C), one-way ANOVA with Bonferroni's multiple comparison test (B). (D) More representative images of amyloid-β plaques in APP/PS1 mice immunostained with Aβ antibody 6E10 in coronal mouse brain cryo-sections. (TIF) [file pone.0151147.s006.tif]
