# Supplementary material for: Traditional Chinese Nootropic Medicine Radix Polygalae and Its Active Constituent Onjisaponin B Reduce β-Amyloid Production and Improve Cognitive Impairments
Source: PLoS One. 2016 Mar 8;11(3):e0151147. doi: 10.1371/journal.pone.0151147 (PMC4782990; doi:10.1371/journal.pone.0151147)
Supplement: S1 Protocol — (DOCX) [file pone.0151147.s007.docx]

**Supporting information**

**Supplementary materials and methods**

**Preparation and fractionation of extracts from RAPO**

**Plant materials**

The dried root of *Polygala tenuifolia Willd.* was supplied by Jiangyin Tianjiang Pharmaceutical Co., Ltd, Jiangyin, Jiangsu Province, China, in December 2012. A voucher specimen (20121201) was deposited in the Institute of Traditional Chinese Medicine & Natural Products, Jinan University, Guangzhou, China.

**General procedure**

Column chromatography was performed with Diaion HP-20 (Mitsubishi-Chemical, Japan), and SKP-10-8300 (Jinan Bona Biological Technology Co., Ltd, Shandong, China). Analytical HPLC was performed on a Shimadzu LC-6AB series pump equipped with a UV detector and a reversed-phase C18 column (Gemini, 5 μm, 250 × 4.6 mm; Phenomenex). All solvents used in column chromatography were of analytical grade (Tianjin Damao Chemical Plant, Tianjin, China).

**Extraction and isolation**

The dried rhizomes of *Polygala tenuifolia* *Willd.* (1.5 kg) were finely cut and extracted twice with 60% EtOH under reflux for 2 hours each time. The combined extracts were concentrated by evaporation to yield a residue of 348.0 g (23.3%), which was subjected to column chromatography over HP-20 macroporous adsorptive resins, eluted with water, 30% and 95% ethanol in succession to produce the water fraction (RAPO-1, 130.2 g), and the 30% (RAPO-2, 37.6 g), and 95% ethanol (RAPO-3, 114.4 g) fractions. Fraction RAPO-1 was then loaded on a SKP chromatography column and eluted with water and 30% and 95% ethanol to produce 3 sub-fractions (RAPO-1-1, RAPO-1-2, and RAPO-1-3).

**UPLC-ESI-MS**

The Onjisaponin B was resolved in methanol to produce standard samples with high (500 μg/ml) and low (100 μg/ml) concentrations. The test sample concentration of RAPO-1-3 is 5 mg/ml. The injection volume was 2 μl for all the samples. For quantitation experiment, both LC and MS conditions were optimized for efficient separation and detection. The negative ionization was chosen according to the literature [[1](#_ENREF_1)].

UPLC analyses were performed using an ACQUITY UPLC system equipped with a binary solvent system, an automatic sample manager and a photodiode array (PDA) detector. The chromatographic separation was performed on an ACQUITY UPLC BEH C18 Column (3.0 mm × 150 mm, 1.7 μm, waters, Ireland) at 40 °C. The mobile phases consisted of eluent A (0.1% formic acid in water, v/v) and eluent B (0.1% formic acid in methanol, v/v). These eluents were delivered at a flow rate of 0.4 mL/min with 66% B from 0 to 25.0 min.

The UPLC system was coupled to a hybrid quadrupole, orthogonal time-of-flight (Q-TOF) tandem mass spectrometer (SYNAPT G2 HDMS, Waters, Manchester, U.K.) equipped with ESI. The operating parameters were as follows: capillary voltage of 3 kV (ESI+) or -2.5 kV (ESI-), sample cone voltage of 35 V, extraction cone voltage of 4 V, source temperature of 100 °C, desolvation temperature of 300 °C, cone gas flow of 50 L/h and desolvation gas flow of 800 L/h. In the MS^E^ mode, the trap collision energy for the low-energy function was set at 5 eV, while the ramp trap collision energy for the high-energy function was set at 20-50 eV. Argon was used as the collision gas for collision-induced dissociation (CID) in the MS^E^ and MS^2^ modes. To ensure mass accuracy and reproducibility, the mass spectrometer was calibrated over a range of 50-2000 Da using a solution of sodium formate. Leucine-enkephalin (m/z 556.2771 in positive ion mode; m/z 554.2615 in negative ion mode) was used as an external reference for the LockSpray and was infused at a constant flow of 5 µl/min. The data were centroided during acquisition.

**Cell viability test**

Chemical-treated HEK293/APPswe cells were subjected to the CellTiter-Glo Luminescent Cell Viability Assay (Promega) following the manufacturer’s instructions.

**BACE1 maturation**

HEK293T cells transiently transfected with HA-BACE1 were treated with chemicals for 4 hours and total lysates were analyzed for immature and mature BACE1 levels by western blot.

**Quantitative RT-PCR:**

Quantitative RT-PCR was performed using the primers listed below:

APP sense: 5’-TGGCCCTGGAGAACTACATC-3’;

APP anti-sense: 5’-AATCACACGGAGGTGTGTCA-3’;

APH1A sense: 5’-GGTGGTTGGGAGTCACCTACT-3’;

APH1A anti-sense: 5’-GCGCTGAATACTTCGGAGGG-3’;

APH1B sense: 5’-TGTTTGGTTCATGGCAAGAGT-3’;

APH1B anti-sense: 5’-CAGCAGTCGCATAGAGGGTG-3’;

APLP1 sense: 5’-GGTTCCGTGGTGTGGAGTATG-3’;

APLP1 anti-sense: 5’-GTGGGACCGTTTCCTCTTCC-3’;

APLP2 sense: 5’-TGAGCCTCAAATCGCAATGTT-3’;

APLP2 anti-sense: 5’-CCTGTTGGATCAGGTTCCCAT-3’;

NCT sense: 5’-AATAAAACAGCTCCCTGTGTTCG-3’;

NCT anti-sense: 5’-ACTACGTGGATAACCCCTGTG-3’;

PS1 sense: 5’-GACGACCCCAGGGTAACTC-3’;

PS1 anti-sense: 5’-ACTGACTTAATGGTAGCCACGA-3’;

PS2 sense: 5’-AGTGTGTGATGAGCGGACG-3’;

PS2 anti-sense: 5’-ACTGGGCAGTGTTCTCTCCAT-3’;

Pen2 sense: 5’-TGGAGCGAGTGTCCAATGAG-3’;

Pen2 anti-sense: 5’-GCGCCAGACATAGCCTTTGAT-3’.

**Acceptor photo-bleaching FRET**

Acceptor photo-bleaching FRET was performed as previously reported [[2](#_ENREF_2)]. HEK293 cells were cultured on glass coverslips and transfected. After fixing, cells were washed and mounted on slides. Samples were subjected to acceptor photo-bleaching FRET imaging with a confocal microscope (LAS SP8; Leica) with a 63× /1.40 NA oil objective (Leica). Leica Application Suite Advanced Fluorescence (LAS AF) software was used for image acquisition, registration, background subtraction and data analyses. Photo-bleaching was performed and over 70% bleach efficiency was achieved. Pre- and post-bleach images of CFP and YFP channels were acquired. FRET efficiency was calculated as the percentage of enhancement in donor fluorescence (f) after acceptor photo-bleaching. To correct the FRET efficiency of the photo-bleached region, five non-bleached regions were selected and the average value was considered to be background signal.

**Co-immunoprecipitation assay**

Co-immunoprecipitation (Co-IP) assays of PS1 with BACE1 were performed exactly as reported previously [[3](#_ENREF_3)]. Transiently transfected HEK293T cells were incubated with chemicals for 16 hours and lysed with IP buffer (50 mM HEPES pH 7.4, 150 mM NaCl, 10% Glycerol and 1% CHAPSO) in the absence or presence of the indicated chemicals. The supernatants were incubated with anti-Flag M2 resins at 4 °C for 4 hours. The resins were then washed and eluted with loading buffer before western blotting analysis.

**Split-TEV assay**

The experiment was performed the same as previously reported [[3](#_ENREF_3)]. HEK293MSR cells were seeded and transiently transfected with Split-TEV construct set. Chemicals were added two hours after the transfection. Cells were treated for 16 hours before the measurements of luciferase activity.

**Supplementary references:**

1. Ling Y, Li Z, Chen M, Sun Z, Fan M, Huang C. Analysis and detection of the chemical constituents of Radix Polygalae and their metabolites in rats after oral administration by ultra high-performance liquid chromatography coupled with electrospray ionization quadrupole time-of-flight tandem mass spectrometry. Journal of pharmaceutical and biomedical analysis. 2013;85:1-13. Epub 2013/07/19. doi: 10.1016/j.jpba.2013.06.011. PubMed PMID: 23860503.

2. Wang X, Cui J, Li W, Zeng X, Zhao J, Pei G. gamma-Secretase Modulators and Inhibitors Induce Different Conformational Changes of Presenilin 1 Revealed by FLIM and FRET. Journal of Alzheimer's disease : JAD. 2015;47(4):927-37. Epub 2015/09/25. doi: 10.3233/JAD-150313. PubMed PMID: 26401772.

3. Cui J, Wang X, Li X, Wang X, Zhang C, Li W, et al. Targeting the γ-/β-secretase interaction reduces β-amyloid generation and ameliorates Alzheimer’s disease-related pathogenesis. Cell Discovery. 2015;1:15021. doi: 10.1038/celldisc.2015.21
