# Supplementary material for: Traditional Chinese Nootropic Medicine Radix Polygalae and Its Active Constituent Onjisaponin B Reduce β-Amyloid Production and Improve Cognitive Impairments
Source: PLoS One. 2016 Mar 8;11(3):e0151147. doi: 10.1371/journal.pone.0151147 (PMC4782990; doi:10.1371/journal.pone.0151147)
Supplement: S1 Table — (PDF) [file pone.0151147.s008.pdf]

S1 Table. Main compounds identified in RAPO-1-3 by UPLC-ESI-Q-TOF/MS

Peak	t_R(min)	Identification	$[M-H]^-$ (m/z)		Formula	Error
			Measured	Calculated		(ppm)
**1**	11.36	Onjisaponin A	1703.7333	1703.7328	$C_{80}H_{120}O_{39}$	0.5
**2**	11.67	Onjisaponin L	1847.7754	1847.7751	$C_{86}H_{128}O_{43}$	0.3
**3**	11.88	Onjisaponin G	1455.6478	1455.6432	$C_{70}H_{104}O_{32}$	3.2
**4**	12.56	Onjisaponin F	1587.6890	1587.6855	$C_{75}H_{112}O_{36}$	2.2
**5**	12.99	Onjisaponin W	1731.7245	1731.7278	$C_{81}H_{120}O_{40}$	-1.9
**6**	14.96	Onjisaponin B	1571.6882	1571.6906	$C_{75}H_{112}O_{35}$	-1.5
**7**	15.78	Onjisaponin Ng	1685.7269	1685.7223	$C_{80}H_{118}O_{38}$	2.7
**8**	16.49	Polygalasaponin XXXII	1673.7257	1673.7223	$C_{79}H_{118}O_{38}$	2.0
**9**	17.35	Onjisaponin J	1817.7638	1817.7645	$C_{85}H_{126}O_{42}$	-0.4
**10**	19.03	Onjisaponin Y	1455.6400	1409.6378	$C_{69}H_{102}O_{30}$	1.6
