# Supplementary material for: Traditional Chinese Nootropic Medicine Radix Polygalae and Its Active Constituent Onjisaponin B Reduce β-Amyloid Production and Improve Cognitive Impairments
Source: PLoS One. 2016 Mar 8;11(3):e0151147. doi: 10.1371/journal.pone.0151147 (PMC4782990; doi:10.1371/journal.pone.0151147)
Supplement: S2 Table — (PDF) [file pone.0151147.s009.pdf]

S2 Table. The detailed information of the main peak areas in RAPO-1-3

Peak	t_R(min)	Identification	Area	Estimated content (%)
**1**	11.36	Onjisaponin A	97.3	
**2**	11.67	Onjisaponin L	35.7	
**3**	11.88	Onjisaponin G	83.1	
**4**	12.56	Onjisaponin F	219.6	
**5**	12.99	Onjisaponin W	88.8	
**6**	14.96	Onjisaponin B	292.5	2.4
**7**	15.78	Onjisaponin Ng	53.1	
**8**	16.49	Polygalasaponin XXXII	322.9	
**9**	17.35	Onjisaponin J	249	
**10**	19.03	Onjisaponin Y	112.8	
**Total**			1554.8	12.8
